# Inclusive Approaches for Measuring Demographics of Underrepresented Populations in STEM and Biomedical Research Training Programs

**DOI:** 10.15695/jstem/v7i2.10

**Published:** 2024-02-01

**Authors:** S.E. Paris, A. Dinno, M.C. Marr, A. Raz Link, B.L. Lentz, A. Setthavongsack, S.N. Espinosa, G. Shusterman, J. Abel, K. Harrison, J. Hook, T.W. Alvord, D.M. Richardson, K. Chase, L.K. Marriott

**Affiliations:** 1 Oregon Health and Science University, Portland, OR; 2 Portland State University, Portland, OR

**Keywords:** Evaluation, Measurement, Workforce, Research, Science Education, Historically Underrepresented, Health Inequities, Disparities, Biomedical, Training, Undergraduate, High School, Middle School, Professional Development, Program Evaluation, Equity, Inclusion, Accessibility, Cultural Diversity, Vulnerable Populations, Research Education, Students, Disparity, Disparities, Race, Ethnicity, Sexual Orientation, Gender Identity, Assessment, Disability, Disadvantaged Background, Immigrant, Refugee, LGBTQ+, Sexual Orientation Identity, Language, Reporting, Data Visualization, Adaptive Demography

## Abstract

As federal strategic plans prioritize increasing diversity within the biomedical workforce, and STEM training and outreach programs seek to recruit and retain students from historically underrepresented populations, there is a need for interrogation of traditional demographic descriptors and careful consideration of best practices for obtaining demographic data. To accelerate this work, equity-focused researchers and leaders from STEM programs convened to examine approaches for measuring demographic variables. Gender, race/ethnicity, disability, and disadvantaged background were prioritized given their focus by federal funding agencies. Categories of sex minority, sexual (orientation) minority, and gender minority (SSGM) should be included in demographic measures collected by STEM programs, consistent with recommendations from White House Executive Orders and federal reports. Our manuscript offers operationalized phrasing for demographic questions and recommendations for use across student-serving programs. Inclusive demographics permit the identification of individuals who are being excluded, marginalized, or improperly aggregated, increasing capacity to address inequities in biomedical research training. As trainees do not enter training programs with equal access, accommodations, or preparation, inclusive demographic measures can welcome trainees and inform a nuanced set of program outcomes that facilitate research on intersectionality to support the recruitment and retention of underrepresented students in biomedical research.

## INTRODUCTION

Science, technology, engineering, and math (STEM) training programs have an overarching goal to improve the education and support of their students. This common ground extends across STEM disciplines and training levels (pre-school through graduate school, termed P-20+; Suárez and Beatty, 2022), revealing a shared starting place among STEM programs who want to understand their impact. Yet, impact can be interpreted differently based on the perspectives of the involved individuals and/or groups (e.g., students, programs, funders), shifting the core questions asked across audiences and settings (Table 1). Evaluation is often a key part of STEM training programs, yet confusion exists around when and how to ask demographic questions. This confusion is due to the nuance and complexity of demographics, which can perplex new and experienced educators alike (Mekinda et al., 2022). Demographic categories have evolved over time, reflecting the shifting identities and language of our students and larger population (Krieger, 2012; Kronk et al., 2022). Even when intentions are good, the timing and language used when asking demographic questions can perpetuate marginalization, stigma, and harm. Further, some demographics are not asked, which can result in functional erasure of identities that reduce inclusion in STEM training programs. Our shared objective to enhance STEM education and provide better support to students creates a unique opportunity for us to work collaboratively. Although readers and those invested in this cause may come from different backgrounds and have different perspectives, their collective interest in supporting the training of our biomedical workforce should foster a sense of commonality for building the trust needed to achieve our common goal.

### Settings and Contextual Factors.

With our collective, overarching goal of improving the education and support of students in our STEM training programs, this manuscript aims to equip STEM program leaders with appropriate language and operationalized structures to improve their demographic data practices. Recognizing that demographic questions are often politicized, we face these issues squarely by providing persuasive justifications that STEM program leaders can deploy when facing opposition from their teams or communities. We fully acknowledge that the geopolitical setting of a STEM program’s location, its training duration, student engagement formats, as well as audience ages served by the STEM program will influence demography practices, requiring an inherent need for flexibility. It may not be safe for students to disclose demographic information in some settings or locations. Thus, permitting students to maintain autonomy and sovereignty over their demographic information is paramount. Unequivocally, readers of this article need to know that demographic data collection requires an iterative approach, as there is no one “right” method because identities and the language used to describe demographics naturally shift over time. Thus, demographic tools will always be incomplete and searching for a comprehensive static tool is doomed for failure. Instead, STEM programs need to be cognizant that demographics reflect an iterative ‘workin-progress’ as we learn how to be more inclusive of populations who are being excluded or marginalized (Krieger, 2012). What may have been considered the best method in the past may not necessarily be appropriate or accurate in the present or future. Demographics should be adaptive to reflect lived experiences within social contexts; therefore, changing contexts will reveal new identities and reflect changes in how demographic groups refer to themselves. With a goal of progress over perfection, STEM training programs can deploy demographics with an aim of supporting and protecting their trainees, recognizing that we are all learning, and that individuals or programs may be at different stages of incorporating diversity, equity, inclusion, and accessibility practices in their STEM training programs.

### STEM Landscape in the Context of Federal Funding Agencies.

The National Institutes of Health (NIH) and the National Science Foundation (NSF) are the two primary funders of STEM-related research in the United States (U.S.). Their strategic plans emphasize training of a diverse biomedical workforce that bridges student educational levels and STEM disciplines to enhance global leadership, accelerate breakthroughs, and support a strong economy and national security (National Institutes of Health, 2021, National Science Foundation, 2022). The National Science and Technology Council’s (NSTC) Committee on STEM Education (CoSTEM) aims to ensure that all U.S. residents have equal and consistent access to high-quality STEM education; they created a five-year strategic plan that identified three main goals: 1) build strong foundations for STEM literacy, 2) increase diversity, equity, and inclusion for those historically underrepresented and underserved in STEM, and 3) prepare the STEM workforce for the future (Committee on STEM Education of the National Science and Technology Council, 2018; Office of Science and Technology Policy, 2021).

In the United States, the NIH and NSF funding agencies define which demographic groups are considered under-represented (National Center for Science and Engineering Statistics, 2023; National Institutes of Health, 2019), with differences across the funding agencies described in Appendix A. Due to these two agencies providing the majority of funding for STEM training, their defined groups are widely used and replicated by other STEM programs and organizations. When aligning solely with federal reporting, STEM programs may be complicit in their data collection practices without realizing that they are excluding or marginalizing demographic groups who are also underrepresented in STEM, thereby perpetuating STEM inequities for individuals that remain unmeasured and uncounted. In federally funded STEM training efforts in the U.S., diversity often refers to increasing the representation of students from racial and ethnic minorities, low-income backgrounds, as well as disabled students who are underrepresented in health and science professions (Boekeloo et al., 2015; Duffus et al., 2014; Valantine and Collins, 2015). While women are acknowledged as underrepresented in NSF-funded research (National Center for Science and Engineering Statistics, 2023; Appendix A), individuals identifying as LGBTQIA+ (i.e., lesbian, gay, bisexual, transgender, queer, intersex, asexual, etc.) remain formally excluded by both federally funded agencies despite also being underrepresented (Cech and Waidzunas, 2021; Freeman, 2018, 2020; Appendix A). Fortunately, recent Executive Orders have called for improved efforts to advance equity within demographic data collection “with respect to race, ethnicity, religion, income, geography, gender identity, sexual orientation, and disability” (White House 2021a, 2021b, 2021c, 2021d, 2022, 2023a, 2023b; Appendix A). These efforts aim to improve data disaggregation and demographic data collection within STEM, important for enhancing training of a diverse STEM workforce within federal strategic plans. These efforts are likely to accelerate scientific breakthroughs, as historically underrepresented scientists produce higher rates of scientific innovation, yet their careers are more likely to end prematurely and their research undervalued by the scientific community (Hoppe et al., 2019; Hofstra et al., 2020). Improving efforts to identify and support diverse groups of scientists throughout their STEM training is likely to improve recruitment and retention of a diverse scientific workforce.

### Enhancing Diversity in STEM Requires Thoughtful Effort.

It is important to remember that scientists and STEM program staff may not have prior diversity training and that their own training experiences could be highly variable. Similar to scaffolded approaches used by STEM programs when working with students across training levels, this article applies a scaffolded approach that supports new and experienced learners to grow in the area of inclusive demographics. Given this consideration, we will establish and use common terminology throughout the article, as defined by researchers who specialize in the subject. Specifically, the OHSU-PSU School of Public Health’s Diversity, Equity, and Inclusion Committee (2022) defined diversity “as the prioritization of representation by historically oppressed and marginalized groups, both in practices shaping the composition of our student, administration, and faculty bodies and structures, and in the centering of perspectives from them in the routine course of our collective work.” This committee reminds us that diversity describes a group characteristic, not an individual, and that while operationalizing demographic data as ‘lists’ are of “instrumental and practical value, they offer little in regards to productive value if not coupled with explicit considerations of presence and representation, and critical examination of historic and present factors that may lead to certain expressions of diversity.” Simply put, they remind us that the “pursuit of ‘checking off’ official social categories on such lists is fundamentally misguided in the absence of attention directed to why the items are on the list to begin with; compliance does not confront power in the service of oppression and marginalization” (OHSU-PSU SPH DEIC, internal document, 2022). In short, enhancing diversity requires a thoughtful process that is fundamentally iterative to be more inclusive of the students in our training programs.

### Goals for Inclusive Demographics in STEM Training Programs.

Inclusive demographics (1) invite and welcome individual representation, (2) represent population diversity, (3) provide the necessary lens to address population inequities and strive for equity, and (4) direct individuals to group-specific resources (e.g., referring students with disabilities to disability resource centers for securing academic accommodations). Recognizing that students, programs, and funders may have different goals and needs for demographic data (Table 1), this article presents relevant use cases that offer guidance, recommendations, warnings, and uses for demographic data for supporting diverse groups of STEM students. With an eye towards growth and progress, we do not intend to provide an exhaustive list of demographic groups, but rather offer practical suggestions for improving intentionality around collecting and using demographic data. Ultimately, inclusive demographic data practices permit the identification of individuals who are being excluded, marginalized, or improperly aggregated (Fernandez et al., 2016; Morrison et al., 2021), enabling STEM programs to address inequities in training. As trainees do not enter our programs with equal access, accommodations, or preparation, inclusive demographic measures can help capture a nuanced set of outcomes that facilitate research on intersectionality between demographic variables, including how better to retain underrepresented students in biomedical research (Duffus et al., 2014; Estrada et al., 2016; Hinton et al., 2020; Valantine and Collins, 2015; Valantine et al., 2016).

### Goals and Organizational Overview.

The key goals of this manuscript are designed to support the unique needs and interests of students, programs, and funders. For students, the goals involve describing how demographics can be used to invite and welcome individual students, making them feel proud and seen; suggesting how data can guide student support; and offering recommendations for data transparency to build trust with trainees. The goals for programs include offering operationalized phrasing as a starting point for program use, recognizing an iterative process to reflect shifting realities of identity and language; providing guidance around recommendations and concerns for inclusion and data use; and encouraging flexibility in data collection across settings to reduce harm and stigma. For funders, the manuscript describes how demographics can inform approaches for enhancing recruitment and retention; inform categories for data collection across grantees; and provides recommendations for aligning research and training across funder initiatives to facilitate transparency and accountability around policies for what is done with demographic information.

Our results section shares settings and use cases that shape demographic data considerations, offering fundamental recommendations that serve as a starting place for being more inclusive with demographic data practices. We share warnings, concerns, and potential pitfalls in the pursuance of inclusive demographic practices, with applied uses for demographic data across training settings. Opportunities for readers to ‘level up’ in their demographic data practices are shared to improve data collection, management, use, and reporting. We offer recommendations for continuing education and training around demographic data practices as well as detailed considerations for inclusive measurement across demographic groups. Our paper concludes with a discussion of recommendations for further improvements, limitations concerning safety and protections, and future directions for this work.

## METHODS

### Setting.

The co-author team comprises STEM trainees, STEM programmatic staff and administrators, STEM evaluators, as well as faculty and associate deans who work closely with STEM-specific programs and/or whose research aims to improve diversity, equity, inclusion, and accessibility across a broad spectrum of student ages and disciplines. Positionality statements of the co-author team are described in Appendix B (Holmes, 2020). Together, our co-author team identified essential questions and considerations for understanding program impact across perspectives of students, programs, and funders (Table 1). The programmatic perspectives and STEM audiences used to shape this manuscript are described in Table 2, though co-authors do not purport to represent the official positions of these programs. Instead, they offer frames needed to support others who work with similar programs or student populations nationwide. The broad representation of our co-author team across student ages and disciplines is intended to enhance the generalizability of recommendations and potential reach to STEM programs.

A subset of this manuscript’s co-authors were instrumental in the iterative development the federally-funded STEM Assessment and Reporting Tracker (START), an informatics platform intended to help schools and programs better understand the STEM development of their students (Paris et al., 2021; NIH SEPA 5R25GM129840, PI: Marriott). In the process of defining demographics to be used within START, it became clear that existing data collection practices (i.e., variables used in federal progress reports) were inadequate to describe the complexity and intersectionality of student identities. As such, expertise from equity researchers and background literature were sought to inform demographic data practices. This article incorporates the insights gained over a span of five years while building START. Our prior work described the prevalence of demographic practices across youth-focused cancer research training programs as well as programmatic desires for ongoing training (Mekinda et al., 2022). This article responds directly to those requests, offering phrasing and training to help programs in their demographic data practices.

### Qualitative Feedback and Analysis.

Lessons learned and operationalized demographic phrasing were shared at regional presentations (Paris et al., 2022a, 2022b, 2022c; Appendix C), which aided in curating audience questions and concerns via Google Jamboard, Zoom chat, and/or orally. Presentation attendees were informed that their anonymous perspectives would be used to guide future work (OHSU IRB #22889). In January 2023, and monthly thereafter, co-authors met to summarize efforts and share considerations important for STEM programs across invested parties’ perspectives and interests. The hybrid meetings were recorded, transcribed, and coded for themes using Taguette (version 1.4.1). Coded data were exported to Microsoft Excel (version 2016) for secondary analysis, with the codebook and summarized tables shared back with co-authors, a process termed “member checking” (Birt et al., 2016). The resources recommended by presentation attendees or co-authors for learning more about inclusive demographic practices are described in Appendix D, offering readers a scaffolded approach for learning more and improving demographic data practices.

## RESULTS

### Settings and Use Cases Shaping Demographic Data Considerations.

In the January 2023 co-author meeting, it was quickly realized that the co-author team prioritized different needs and goals for demographic data practices based on their individual roles (e.g., direct work with students, program administration, evaluation, as equity-focused researchers, etc.). Table 3 highlights use cases that illustrate distinct yet interrelated perspectives offered by our co-author team that can serve as a roadmap for recommendations and concerns that may be relevant to individuals or groups facing similar demographic data considerations. Thus, the considerations for recommendations, operationalized phrasing, and warnings related to demographic data are highly influenced by the perspectives of the involved parties as well as the contextual settings of the particular program, such as the geopolitical landscape. Ultimately, these perspectives reinforce that flexibility is needed—for programs and students alike—as the conditions for data collection, reporting, and data usage will vary based on the perspectives of those involved and STEM program setting.

### Fundamental Recommendations.

In an effort to help STEM programs meet their common goal of improving STEM education and support for students, we offer eight fundamental recommendations that serve as a starting place for being more inclusive with demographic data practices:

1. **Expect changes over time**. Demographic tools will always be incomplete, so processes must enable adaptation over time to reflect shifting identities and languages that are more inclusive of populations being excluded, marginalized, or improperly aggregated (Table 4). In addition to changes in data collection tools, the individual identities of students may also change throughout their participation in a program. Measuring demographics over time accommodates shifting identities and enables students to disclose identities based on their comfort with your STEM program or changes in life experience.
2. **Demographics make trainees feel seen, welcomed, and invited**. Adding recognizable language around self-identities makes trainees feel more welcomed, more invited, and more willing to participate. Demographics should help trainees feel seen and proud of their disclosed identities. Examples of data uses are described in Table 5.
3. **Remember that data collection and reporting are different things**. Since the goal of inclusive demographics is to help trainees feel welcome, remember that what is asked can differ from how it is analyzed/reported. Taking collected data and combining them into an overarching analytic category for reporting can be okay. The raw data still exist and matter. See data practices described in Table 6 for recommendations and guidance.
4. **Flexibility in demographic practices is essential.** While a goal for comparable data exists among evaluators and funders, flexibility must remain to support programs of different formats, durations, and audience ages. Flexibility also protects students’ privacy across geopolitical settings and builds in time to establish trust with trainees before asking them to disclose personal information. Specific program audiences should also guide the approach (e.g., “I work in a program where the difference between a Samoan and a Marianas Islander is really important, because those are two really different cultures. But another program may not need those specifics [in lieu of] other relevant information. Demographic checkoffs are not going to be consistent across programs or studies”).
5. **Lean on phrasing developed from others.** Leverage approaches described in the literature as a starting place, rather than creating your own phrasing that can “other” a particular group. Poor phrasing can create unintentional stigma and harm among trainees (Table 4 describes warnings and potential pitfalls). Operationalized phrasing can support checkbox approaches (for large scale data collection) and free text responses (for smaller scale data collection), enabling flexibility and a starting place for language (Appendix C).
6. **Look for populations missing or excluded.** While previously-developed phasing serves as a starting point, programs should be wary of taking categories at face value, because groups may be missing (i.e., LGBTQIA+), excluded (i.e., non-federally recognized Indian tribal members, refugees, immigrants), or improperly aggregated (i.e., Middle Eastern/North African, Asian). Be an advocate rather than perpetuating exclusion of already marginalized groups.
7. **Build trust by being transparent in your data use practices.** There are inherent power dynamics with demographic data collection in terms of what is asked and when. Build trust with students by describing why you’re asking, what will be done with the data, and who has access to the data. See more in data practices (Table 6).
8. **Seek training and speak up.** Practice a growth mindset by recognizing that knowledge of inclusive data practices can be learned over time. Seek training, talk with experts, and dive deeper to improve demographic data practices that are more equitable, inclusive, and supportive of a diversity of identities. Remember that “promoting diversity requires explicit acknowledgment of historic and current mechanisms of oppression and marginalization,” which takes both time and active work (OHSU-PSU School of Public Health’s Diversity, Equity, and Inclusion Committee, internal document, October 12, 2022). Recognize that your team or community may be at a different place than you are; proceed anyway and be a champion for change.

### Warnings, Concerns, and Potential Pitfalls.

Given the complexities and iterative processes of demographic data collection, those interested in collecting more inclusive demographic data may feel ill-prepared to proceed. They are not alone. Many individuals involved in STEM programs may have strong expertise in a STEM-specific area (e.g., immunology, computer programming, cancer), yet be unfamiliar with student evaluation or inclusive demographic data practices. We encourage readers to “lean in” anyway, as growth and comfort rarely co-exist (Rometty, 2023), meaning that growing professionally in an area can often be associated with discomfort as we learn a new area. As with other collaborative teams in science, members of your program’s community can push each other to see new perspectives, share resources, and improve practices. We offer a set of warnings and potential pitfalls to guide your collaborative work (Table 4).

### Applied Uses for Demographic Data Across Training Settings.

Despite warnings and concerns (Table 4), there are many benefits for including and using demographic data to support students and improve STEM programs. Table 5 offers ideas for using demographic data with trainees, such as welcoming students, encouraging diverse perspectives, providing tailored support, and directing students to specific resources. In the practice of using data to improve training, it is imperative that STEM programs be transparent about their practices and procedures (Table 6).

### Leveling Up Demographic Data Practices.

Demographic data are inherently personal, even when collected anonymously. The recognition that data collection can cause stress and potential harm to students reinforces the necessity for ethical and responsible data practices. While granular data collection can be used to welcome and recruit underrepresented students, programs need to be mindful of the specifics of their practices, such as when demographic data are asked and how frequently. If our collective goal is to improve the recruitment and retention of historically underrepresented students, STEM programs need to be reflective about their data collection practices and whether their evaluation informs for whom their programs are working. Recommendations for improving data collection, management, use, and reporting are described in Table 6.

### Training Is Needed Around Demographic Data Practices.

By their nature, inclusive demographic data practices are iterative and adaptive, and will require continuing education and training. As the geopolitical setting of a program can influence the practices and perspectives of all interested parties, we recommend STEM programs incorporate demographic discussions into programmatic meetings, and that training occurs at individual, programmatic, and national levels. An important aspect of this iterative engagement will be conversations to share effective solutions learned across programs, and to elevate concerns and pitfalls. STEM programs have previously cited a desire for ongoing training in demographic data practices (Mekinda et al., 2022), though there is currently not a single resource, person, or national group that can guide questions about demographic representation and advise practices. Inclusive demographics support could be an important federal initiative that could minimize problematic language across demographic survey questions, analysis, and reporting. It could also help to harmonize data collection efforts across federally funded STEM programs needed for comparability.

### Considerations for Inclusive Measurement Across Demographic Groups.

Executive Orders, such as #13895, called for federally funded programs to assess “equity with respect to race, ethnicity, religion, income, geography, gender identity, sexual orientation, and disability” (White House, 2021a). Our prior work found inconsistencies in demographic data practices of youth-focused cancer research training programs (Mekinda et al., 2022), highlighting opportunities to support STEM programs in how to be more inclusive in their demographic practices. For example, while race and ethnicity were consistently collected by all measured cancer research training programs (15/15; 100%), other demographic variables were less consistently measured, such as gender (80%), disability (71%), and sexual orientation (7%). Viewed together, two thirds of the NIH-funded cancer research training programs (67%) measured underrepresentation using broad NIH categories (i.e., race/ethnicity, disability, and socioeconomics in some form), though only half (53%) explicitly referenced using the revised criteria (National Institutes of Health, 2019), which uses seven variables to categorize disadvantaged background. Recognizing the challenges and complexities, we offer operationalized phrasing and considerations (Appendix C) that can provide STEM programs with a starting place for their demographic data collection, recognizing that the groups we describe briefly below are not exhaustive of the populations that should be measured.

#### Race and Ethnicity.

Racial and ethnic groups are underrepresented in biomedical research, including Black or African Americans, Hispanic or Latino Americans, American Indian or Alaska Natives, as well as Native Hawaiian or other Pacific Islanders (National Institutes of Health, 2019). Indeed, fewer students of color enroll in STEM majors when entering college and leave STEM majors at higher rates (Gaston Gayles et al., 2018). Federal reporting forms are highly aggregated, lumping students across racial and ethnic groups. The specifics of the trainee’s identity become masked without data disaggregation. For example, “Asian” includes individuals from across the world’s largest continent without consideration to the resources where the individual grew up (i.e., Southeast Asia). Likewise, Middle Eastern and North African (MENA) individuals are often categorized as White on U.S. Census forms (Office of Management and Budget, 2019; Federal Register #58782, 1997), yet represent diverse geographies and backgrounds who remain marginalized (Kayyali, 2013; Maghbouleh et al., 2022). Although refugees or immigrants who have been displaced by conflict may encounter obstacles in accessing and participating in STEM programs and resources, it remains a challenge for STEM programs to quantify these individuals as no demographic category for them exists within disadvantaged backgrounds (Appendix A). Further, many Tribal affiliations, Asian, and Pacific Islander sub-groups are masked. Greater resolution in data collection practices can help to identify health and STEM disparities in subpopulations, guide development of culturally specific and accessible services, and guide equitable allocation of STEM-related resources to address inequities. The Oregon Health Authority’s Race, Ethnicity, Language, and Disability (REALD) offers a validated tool for collecting disaggregated demographic information on race and ethnicity, as well as language and disability (McGee, 2020). REALD’s 34 racial and ethnic identities map to current NIH and NSF demographic categories (Appendix C) and include a write-in option for individuals to self-describe identity if not included in the list, which helps to track emergent populations. This tool offers a great way for students from diverse racial and ethnic groups to be seen and welcomed.

#### Disability.

As with all demographic categories, adapting language to better reflect diverse identities and experiences of individuals with disabilities promotes inclusivity and helps to create a more welcoming and equitable environment. This is exemplified by Rosa’s Law, which removed outdated and offensive slurs previously used by federal, health, education, and labor policies to describe people with intellectual disabilities (Degeneffe and Terciano, 2011). Individuals with disabilities may experience discrimination and challenges living in a society not built for them, which can impact their health and quality of life (Hammell, 2015; Robertson, 2010), and how they engage in STEM education (Steele and Wolanin, 2004). As disability can exist on a spectrum and the impact of functional limitations may not be visible to others, careful consideration needs to be given to how to collect and measure disability data. Those with “invisible” disabilities (e.g., chronic pain, cognitive deficits, diabetes) often have more trouble accessing support services as they have the extra burden of “proving” their disability to others, which often leads to greater suffering, and can exacerbate their disability (Davis, 2005). NIH defines individuals with disabilities as those with a physical or mental impairment that substantially limits one or more major life activities (National Institutes of Health, 2019). Applying the definition or understanding what is meant by functional difficulties may be challenging for students replying to demographic questions. The REALD instrument (McGee, 2020) assesses functional health and service differences, including hearing, vision, movement, communication, daily living, cognition, as well as mental and emotional health (Appendix C). Along with age-appropriate question prompts, the REALD instrument provides concrete examples in everyday life, and allows STEM programs to provide specific accommodations and resources, which could be particularly helpful for postsecondary students. Although supports for students with disabilities are mandatory in grades K-12 (e.g., an Individualized Education Program (IEP) enforced by the Individuals with Disabilities Education Act (IDEA)), colleges and universities have no similar mandatory process, hence the burden of finding and implementing supports for a successful educational outcome are placed on the students with disabilities (Newman et al., 2011). Adopting a neurodiversity perspective that views neurodivergent individuals and individuals with neurological-developmental disabilities through the lens of human diversity allows researchers and educators to emphasize individuals’ strengths, gifts, and talents (Robertson, 2010). While specifics related to neurodiversity are not explicitly addressed, REALD can be used to encourage strengths-based conversations about strategies used by students for succeeding in STEM programs. REALD also has the advantage that results can be directly mapped to current NIH demographic categories (Appendix C). We encourage STEM programs to be trauma-informed in their approach (Isobel, 2021) and consider neurodiversity when incorporating social support into programs, as students can share strengths-based approaches and strategies for succeeding in STEM education with each other.

#### Disadvantaged Background.

Disadvantaged background often describes the socioeconomic or environmental conditions that impact access to a student’s STEM education or training environment. Disadvantaged background definitions have evolved over time, with NIH issuing definitions related to socioeconomics and class in 2018, which were changed in 2019 to a multi-step process (National Institutes of Health, 2018, 2019). Specifically, NIH defines individuals as being from a disadvantaged background if they meet two or more of the following seven criteria: 1) experienced past or present homelessness; 2) previously or presently in the foster care system; 3) eligibility for the Federal Free and Reduced Lunch Program for 2+ years; 4) first generation college student status (i.e., parents or guardians without a bachelor’s degree); 5) eligibility for Federal Pell grants; 6) received WIC as a parent or child; and 7) grew up in a rural area as defined by either Health Resources and Services Administration (HRSA) or Health Professional Shortage Area (HPSA) locations (Centers for Medicare and Medicaid Services, n.d.; Health Resources and Services Administration, n.d.).

When using these questions with students, prior work found that students underreported their disadvantaged background; both first generation college student status and rural area residency were underreported when verified (Marriott et al., 2022; Huerta et al., 2022). In open prompts, a student reported a limitation of HPSA and zip codes, as they “moved a lot/[were] homeless.” Such limitations would also be seen for other students who experienced frequent moving (e.g., military family background, immigrants, refugees). While medical codes for housing instability (i.e., ICD-10 code Z59.81) could be applied in some cases (Rollings et al., 2022), these codes are not federally recognized and do not describe the full range of students’ lived experience with disadvantaged background. Therefore, how STEM programs incorporate these variables into their efforts and whether they verify student data against other variables (which can take time) becomes a judgment call for programs. Ultimately, these distinctions can impact student eligibility for training programs or scholarships as well as data accuracy.

#### Sex Minority, Sexual Minority, and Gender Minority (SSGM).

Informed and respectful discussion around the issues that affect sex minority, sexual minority, and gender minority (SSGM) individuals begins with a mutual understanding of the distinctions between sex, sexual orientation, and gender.

- **Sex** is a categorization that refers to biological traits such as the appearance of genitalia, chromosomal structure, hormone levels and cycles, and secondary anatomical characteristics in addition to legal sex designation (Morrison et al., 2021). Historically, sex has been assigned at birth based on the appearance of external genitalia using binary male/female sex categories, although intersex, third sex, and unspecified (‘X’ and ‘U’) categories bluntly capture bothness, betweenness, and neitherness. Sex minorities are those whose biological sex does not align with typical binary classifications.
- **Sexual orientation** refers to the characterization of an individual’s own emotional and sexual attraction to others (e.g., gay, lesbian, bisexual, pansexual, straight, heterosexual), and also their location along continuums (e.g., asexual to sexual, aromantic to romantic, etc.; Appendix C). An individual’s sexual orientation can only be ascertained by asking them directly—i.e., sexual orientation cannot be assumed based on an individual’s choice of partner(s) or sexual behaviors (Bamberger and Farrow, 2021). Some individuals may experience fluctuations in their sexual orientation and identity throughout their life. Sexual minorities are those whose sexual orientation does not fit within the heterosexual norm, and can include people who identify as gay, lesbian, bisexual, pansexual, asexual, or any other non-heterosexual orientation.
- **Gender** is a “socially constructed categorization defining roles in social relationships, typed behaviors, and self-identity. Gender expression refers to the multitudes of ways in which people present themselves (masculine, feminine, androgynous, etc.)” (Morrison et al., 2021). Gender minorities are those not occupying the categories cisgender woman or cisgender man, and can include transgender, nonbinary, genderqueer, or any other nonconforming gender.

Acknowledging these distinctions, and the ways power operates differently among them, provides the context for understanding SSGM differences. The acronym SGM (sexual and gender minorities) may be used when discussing considerations specific to sexual and gender minorities, without the inclusion of sex minorities (e.g., when referring to sexual orientation and gender identity (SOGI) demographics). Adolescents navigating changes in their sexual orientation or gender identity may be unsure of how to answer SOGI questions, however it is still important to ask. In an article published in the Journal of the American Medical Informatics Association, Goldhammer et al. assert that in the United States, “an estimated 9.5% of adolescents aged 13–17 years old identify as sexual and gender minority, and children as young as 2 or 3 years old may declare a transgender or gender diverse (TGD) identity” (2022). The SOGI measures we recommend not only accommodate a broad range of identities, but also those who are uncertain, prefer not to answer, or don’t understand what the questions are asking. Appendix C provides additional definitions and operationalized phrasing for demographic categories. Gender identity and sexual orientation identity are specifically described below.

#### Gender Identity.

The NSF, but not NIH, consider women to be underrepresented in STEM (Appendix A). Yet, those who identify outside the gender binary as well as other SSGM individuals remain hidden and are not federally recognized as underrepresented despite facing significant marginalization, barriers, and exclusion (Campbell-Montalvo et al., 2022b; Freeman, 2020; Kronk et al., 2022; Palmer et al., 2022). Conflating gender and sex in research practices excludes entire populations whose physical characteristics do not fit neatly within the categories female or male (i.e., sex minorities) as well as those whose gender identities do not fit within the woman/man binary or are transgender (i.e., gender minorities; Ashley, 2022; Heidari et al., 2016). Additionally, when gender discussions are framed within the dichotomous social construct of cisgender and transgender, the gendered experiences of intersex individuals and those with nonconforming genders who do not consider themselves transgender are overlooked (Ashley, 2022; Morrison et al., 2021). To address this inadequacy, Ashley (2022) proposes the adoption of a new term, gender modality, which “refers to how a person’s gender identity stands in relation to their gender assigned at birth” (see Appendix C). Gender modality encompasses transgender and cisgender identities, while also inviting the expression of additional terms that capture people’s varied experiences in relation to their gender identity and gender assigned at birth (Ashley, 2022).

The erasure of sex and gender minority groups makes it impossible to collect accurate data for these populations (Morrison et al., 2021), essential for STEM equity described in federal strategic plans. A critical issue highly relevant to STEM training is how transitioning gender before college affects FAFSA and selective service registration (Prescott, 2017). The federal government’s failure to address these issues delays federal aid for transgender and gender-minority students, and in some cases, prevents them from accessing financial aid necessary for higher education (Prescott, 2017). While demographic questionnaires frequently ask about gender, there is less consistency about how questions are asked (Heidari et al., 2016). One of the most pertinent implementations for consistency across gender measures is to disentangle sex and gender. When survey measures lack resolution, researchers often make assumptions about gender, which inherently undermines the validity and reliability of the survey results, and hinders researchers’ abilities to draw meaningful conclusions. In the context of inclusive demographic measurement, STEM programs should measure gender and sex as independent variables, which allows researchers to more accurately capture the nuances of gender and avoid making assumptions based solely on an individual’s sex. Open-ended prompts are most supportive of gender and sex diversities and can be qualitatively coded. If coding an open-ended prompt is not possible (i.e., in large studies, programs, organizations), we suggest using the two-question approach from Morrison et al. (2021), or questions two and three from the measure currently being developed by the Oregon Health Authority Office of Equity and Inclusion (2022), both listed in Appendix C. In an effort to prioritize the needs, preferences, and experiences of the LGBTQIA+ community, the National Academies of Sciences, Engineering, and Medicine, the Institute of Medicine, and The Joint Commission all recommended collecting and documenting SOGI demographics in research, health, and/or administrative settings (National Academies of Sciences, 2022; Grasso et al., 2019). Similarly, in their technical report, Improving Measurement of Sexual Orientation and Gender Identity Among Middle and High School Students, Temkin et al. (2017) suggest that collecting data in educational settings could help to support the development of programs, practices, and policies that promote healthy development and safe, supportive environments.

#### Sexual Orientation Identity.

Sexual orientation exists on a spectrum and is independent from gender identity, though the two are often conflated in research studies (Heidari et al., 2016). Measuring sexual orientation separately from gender and sex provides a more accurate representation of SOGI populations. Sexual orientation is not considered underrepresented according to federal definitions (Appendix A) and many STEM training programs do not ask about sexual orientation (Freeman, 2020; Marr et al., 2022; Mekinda et al., 2022). However, research shows that LGBTQ+ populations face significant health disparities and discrimination (Gonzales et al., 2016; Kronk et al., 2022; Samuels et al., 2021). Not only are LGBTQ+ individuals underrepresented in STEM (Cech and Waidzunas, 2021), but they are more likely to be harassed as well as face stigma, discrimination, and bias (Campbell-Montalvo et al., 2022a; Cech and Waidzunas, 2021; Freeman, 2018; Freeman, 2020; Hughes, 2018; Marr et al.,2022; Palmer et al., 2022). The distinct barriers that LGBTQ+ trainees face because of their sexual orientation and/or gender identity impede their career advancement despite making considerable contributions to STEM fields (Cech and Waidzunas, 2021; Marr et al., 2022).

We fully recognize that some STEM programs may be hesitant to ask about SOGI demographics, due to student age, privacy considerations, or the program’s geopolitical setting. We encourage STEM programs to be mindful of student safety and privacy while making space for students to be their authentic selves in their programs. As LGBTQ+ students face significant barriers and are underrepresented in STEM, programs should work diligently to improve the STEM training environments for these students, incorporating partnerships with identity-focused organizations that can improve support for sexual and gender minority students, particularly those who have intersectional identities with underrepresented racial and ethnic groups (Campbell-Montalvo et al., 2022a). SOGI diversity, protections, and freedom of expression are internationally recognized, with the International Panel of Experts in International Human Rights Law on Sexual Orientation and Gender Identity (2017) providing guiding principles for equity and inclusion. In sum, STEM programs should improve data practices and training environments to prevent academic exclusion based on SOGI.

#### Language.

Within the context of inclusive demographics, language is approached from two perspectives. First, and most simply, STEM programs need to be mindful of whether their students and their families can understand the materials being sent to them, including demographic questionnaires and consent forms for participating in STEM training programs. For example, individuals who have English as a second language may need more support or be unfamiliar with terms or phrasing. The REALD (McGee, 2020) provides 2–5 questions that can help STEM programs understand which language(s) are spoken at home to better support communication with individuals and families. For example, STEM programs can use the tool for informing language(s) for translated materials or circumstances where interpreters may be beneficial.

Second, a foundational principle of inclusive demographics is the understanding of how we use language in STEM, which will adapt over time to describe populations more accurately. Individuals can self-identify; however, it is important for STEM programs to understand that the concepts of gender and sexuality are often based on the inherent environment where the individual resides or grew up. For example, while the gender binary may be viewed as dominant in Western culture, there are different gender systems in various North American Indigenous nations, tribes, and communities (Hunt, 2016). In South Sulawesi, Indonesia, the Bugis people recognize five distinct genders (Ismoyo, 2020), reinforcing the need for gender identity measures to include options beyond the binary of man and woman. It also emphasizes that gender identity and sexual orientation should be asked as separate questions rather than being conflated. Further, when translating phrasing for these genders, specific language may be missing, which can take away parts of an individual’s identity or mask it, reinforcing 1) why open-ended prompts are helpful for measuring demographics more inclusively and 2) why data collection and reporting should be viewed as separate things. As a primary goal of inclusive demographics is to make students feel included and welcomed, the open-ended prompts within data collection can support that goal, while reporting can protect privacy while documenting prevalence of SSGM or LGBTQIA+ individuals in STEM training programs.

#### Religion.

Religious or cultural minorities are those who hold religious beliefs or cultural practices that are different from those of the majority in a particular society or region. Religious minorities have reported feeling isolated, excluded, discriminated against, and professionally disadvantaged (Marks et al., 2019). Several White House executive orders aimed at advancing diversity, equity, inclusion, and accessibility for underserved communities recognize religious minorities as underserved due to consistent and systematic barriers preventing them from participating fully in socioeconomic and civic activities (White House, 2021a, 2021d, 2023b). Educational institutions have a responsibility to provide a safe and inclusive environment for students from all religious and cultural backgrounds. Collecting demographic data on religion can lead to the development of inclusive policies, practices, and resources that accommodate religious and cultural diversity, such as flexible scheduling for religious holidays, dietary accommodations, or access to prayer or meditation spaces. Best practices for inclusive religious self-identification should include a broad question that allows those with theistic faiths, spiritual nontheistic beliefs, and nonspiritual worldviews to be captured within one demographic question, while also permitting respondents to select multiple options for those with fluid belief systems or that incorporate multiple worldviews (Hughes et al., 2022; Appendix C).

## DISCUSSION

Inclusive demographics are an essential part of STEM training, as they are necessary for understanding whether all U.S. residents have equal and consistent access to high-quality STEM education defined in federal strategic plans (National Institutes of Health, 2021; National Science Foundation, 2022; Office of Science and Technology Policy, 2021). Underrepresented scientists contribute significantly to research innovation and scientific breakthroughs made within STEM, regardless of whether their demographic groups are federally recognized or not (Cech and Waidzunas, 2021; Hofstra et al., 2020; Hoppe et al., 2019; Marr et al., 2022). While documenting the diversity of STEM scientists and trainees has improved, there remains significant room for growth to be more equitable, inclusive, and consistent (National Academies of Sciences, Engineering, and Medicine, 2023). This article offers guidance around operationalized phrasing and considerations for helping STEM programs improve demographic data practices important for improving their training environments.

The instruments we recommend for inclusive demographic data collection can be found in Appendix C. The measures for race and ethnicity, disability, disadvantaged background, language, and religion are recommended for P-20+ students, although primary school students would likely require parent/guardian assistance to complete the surveys. The measure for gender identity is also recommended for use in P-20+ settings, however, it may require different phrasing if used for children ages 3–13 years (Goldhammer et al., 2022). The sexual orientation identity measure is recommended for use with middle school audiences and older.

Flexibility, adaptability, and student safety remain paramount to this work. We fully recognize that our recommendations to permit flexibility may feel at odds with encouraging disaggregated demographic data collection. Ultimately, this recommendation reflects the perpetual balance between the needs of those involved. While programs, organizations, and funders may want to know whether historically underrepresented students are being increasingly recruited and retained in federally-funded STEM programs, inherent power dynamics must be balanced with considerations around how STEM programs can best support their students. Some students may not feel comfortable or safe disclosing their identities if they believe it may hinder their academics, career advancement, or physical safety. For example, an LGBTQIA+ student whose sexual orientation or gender identity has not yet been revealed to their parent(s)/guardian(s) may not provide accurate information on SOGI surveys for fear of involuntary disclosure of their sexual orientation or gender identity to their parent(s)/guardian(s). As such, demographics should remain voluntary and programs should look for ways to build trust with their trainees, such as being transparent in their policies and use of demographic data.

### Recommendations for Further Improvements.

In an effort to improve inclusive data practices, we highlight the following recommendations, which are also incorporated into Table 7.

1. ***Recognize Intersectionality***. Intersectionality is an analytical framework for examining how race, class, gender, sexuality, ethnicity, nation, ability, age, and other social identities overlap and combine additively to shape people’s experiences (Collins, 2015; Parent et al., 2013). A student will not have a single identity, therefore, programs need to recognize that a student with intersectional identities (i.e., marginalized race/ethnicity, disability, disadvantaged background, LGBTQIA+, etc.) may face additional or synergistic barriers to their STEM training. For example, federal STEM training programs often require full-time participation (i.e., full-time enrollment with academic credits), which can be extremely challenging for students with disabilities, extenuating circumstances (i.e., family deaths, illness), mental health challenges, or those who experience the vicissitudes of disadvantaged backgrounds (e.g., housing instability). Further, as STEM training programs learn about the intersectional identities of students and the barriers they face, programs should elevate concerns and recommendations to funding agencies so STEM inequities aren’t perpetuated and that all U.S. residents can have equitable access to high-quality STEM education. In order to diversify the biomedical workforce and enhance participation from underrepresented groups, we need to be mindful of intersectionality and how social determinants of health influence STEM pathways. We believe that including the SOGI questions in demographic data collection will lead to a clearer understanding of the complexities of gender and identity to better serve LGBTQIA+ communities in STEM training environments. If incomplete data on these communities remain, there will not be an accurate picture of the demographics nor how their STEM experiences may be hindered.
2. ***Use Demographic Data to Improve.*** As STEM programs learn to improve demographic data practices, there may be confusion about how to analyze, report, and use demographic data. Demographics can be used to identify where gaps or inequities may be present, including for intersectional demographic groups (e.g., African American girls, low-income students with disabilities, etc.). Together, data can be used to identify issues and provide students with strengths-based supports and strategies that have been successfully used by others like them. Programs can establish procedures for viewing their data with intersectionality in mind. Programs can also incorporate intersectional demographics as an approach for helping students learn interprofessionally—with, from, and about each other—as they solve complex STEM problems within teams (Health Professions Accreditors Collaborative, 2019). Programs can incorporate feedback loops for soliciting strengths and areas for growth in their programs, which they can use to inform their practices, remediate behavior, or alter specific approaches.
3. ***Incorporate Demographics Into Programs to Provide Identity-Based Mentorship, Student Services, and Supports***. The disclosure of demographics can be sensitive. Given that students have intersectional identities and marginalized students can face higher rates of stigma and bias, it is important for students to have others with whom they can talk about their STEM paths (Marriott et al., 2021). Programs should incorporate the diverse representation of their students into their programs’ decision-making, including at the staff, mentor, and leadership levels. Aligning with Self-Determination Theory’s relatedness (Ryan and Deci, 2020), peer mentors produce strong benefits for supporting a diversity of trainees (Cheryan et al., 2013; Huerta et al., 2022; Walton and Cohen, 2007). Discussions with fellow peers and peer mentors can also help students make informed decisions about their fields and paths (Cheryan, 2012; Else-Quest et al., 2013; Marriott et al., 2021). Informed decisions are essential, particularly for first-generation college students who may be unfamiliar with academic processes and that their FAFSA loans cover only a limited amount of academic credits. Thus, providing demographic groups with specific services, such as academic advising, financial advising, or disability resource center access can help promote equitable achievement in higher education and STEM training among a diverse student body. Likewise, incorporating demographics into mentorship activities can help students navigate experiences that balance STEM with other aspects of their lives (e.g., religion). Inclusive demographics have the potential to enhance relatedness between a diversity of students pursuing STEM education.

### Limitations.

Safety considerations play a crucial role in self-reported demographic data because the reliability of data is contingent upon the accuracy of self-reporting. With the current geopolitical setting of some STEM programs, it is highly unlikely that STEM trainees representing marginalized groups will experience comparable safety and protections nationwide. Considering that students with multiple marginalized identities report higher instances of mistreatment and discrimination (Teshome et al., 2022), respondents cannot be expected to accurately report how they identify their demographics if doing so may compromise their safety. Two primary indicators for safety issues include non-visible identities and positionalities (demographic membership known only when reported) and practical risks tied to identification (e.g., discrimination, harassment, psychological stress, etc.). When disclosing demographic data carries inherent risks, trainees must be strategic in their disclosure, which influences the accuracy of demographic prevalence and representation. As such, the lack of demographic data in an area does not mean that trainees of a demographic group are absent, but rather that they may not feel safe reporting.

STEM programs need to be mindful that individuals may withhold or modify demographic information to protect their safety or well-being. Functionally, this renders challenges to the comparability of demographic data across programs and what works for specific demographic groups. Without safety, accuracy will suffer. The concern within communities around the potential for harm and misuse of personal data is central to how we collect demographic data. Unlike demographics currently recognized by federal funding agencies (e.g., race, socioeconomic status), respondents may not see any practical benefit from self-reporting especially when it carries practical risks. In these circumstances, programs can offer transparency about the benefits of reporting. For instance, if additional demographics qualify individuals for an equity program or benefit, an explanation in respondent-facing materials should be provided. Administering inclusive demographic measures signals the project or program is aware of and comfortable with the existence of diverse demographics, allowing people to self-identify with less perceived risk. As researchers, STEM programs, and funders, we must keep these security concerns in mind and build trust with trainees as we take adequate measures to ensure participant privacy.

### Future Directions.

Inclusive demographics offer a way for trainees to be welcomed and invited into STEM. As we balance the concerns around data safety, disclosure, and limitations to demographic data accuracy, we encourage STEM programs to take a trauma-informed approach to demographic data practices, keeping in mind the contexts in which we ask sensitive questions (Isobel, 2021). These questions have the potential for adverse psychological impacts and can bring up certain aspects of one’s identity in insensitive ways. Thus, trauma-informed practices should be incorporated as universal design principles within all of our STEM training programs, as trainees in our programs may have faced significant challenges and adverse events that are non-visible yet extend beyond disadvantaged backgrounds alone. Offering empathetic and flexible supports for STEM trainees to succeed and thrive in our programs has the potential to improve STEM training environments more broadly while informing approaches for improving the training of a diverse scientific workforce.

## Supplementary Material

Parisetal2024_AppendixA_FedLandscape

Parisetal2024_AppendixB_Positionality

Parisetal2024_AppendixC_Operationalized

Parisetal2024_AppendixD_Resources

## Figures and Tables

**Table 1.** Key questions for understanding program impact for students, programs, and funders.

	Students	Programs	Funders

Core Impact Questions	• Do I feel seen? Do I feel supported? • Am I gaining skills and knowledge in the area? • Do I feel like I could belong within this field? • Am I able to choose my next steps in a timeline that works for me? • If I want to continue, do I have the financial ability to pursue this area?	• Who is our program serving? • How do we tell if our program or intervention is effective? • How do we know for whom our program is working and which groups may need more support or increased representation? • How can we improve and tailor the support we give students to prepare them for next steps? • How do we understand the long-term impact of our work?	• Are programs measuring underrepresented populations in ways that enable robust programmatic comparisons? • Are federally-funded grantees able to address new Executive Orders (EOs) requiring data collection across a broader spectrum of demographics? • Are programs enhancing recruitment and retention for a diverse scientific workforce?
Essential Evaluation Questions	• What do culturally-relevant supports include for students like me? • Which supports are meaningful and effective for students like me? • How do students like me succeed in this area?	• What are the questions our program needs to ask and when? • What are data collection and reporting recommendations and how do these two items differ? • What should our program keep in mind so we can avoid common pitfalls and reduce harm? • How can our team learn and grow to improve inclusivity of our work? • Is our program improving recruitment, retention, or both?	• How do we advise grantees seeking evaluation advice to operationalize the NIH notice of diversity and EOs? • How should programs report demographics in progress reports (RPPRs) and what are targets for change? Who is marginalized? • How can we reduce structural and systemic inequities and biases? • How can funding be directed to initiatives that improve training of a diverse workforce
Manuscript Goals	• Describe how demographics can be used to invite and welcome individual students, making them feel proud and seen. • Suggest how data can guide student support. Offer recommendations for data transparency to build trust with trainees.	• Offer operationalized phrasing as a starting point for program use, recognizing an iterative process to reflect shifting realities of identity and language. • Provide guidance around recommendations and concerns for inclusion and data use • Encourage flexibility in data collection across settings to reduce harm and stigma.	• Describe how demographics can inform approaches for enhancing recruitment and retention. • Suggest categories that should be collected by grantees, plus transparency and accountability around policies for what is done with demographic information • Provide recommendations for aligning research and training across funder initiatives.

**Table 2.** Co-authors work across student training ages, disciplines, and STEM training programs.

Training Audience	Representative Programmatic Perspectives

Middle School (MS) and High School (HS)	**NIH** (NIGMS SEPA, NCI YES); **Non-federal funding**: Portland Metro STEM Partnership
Undergraduate	**NIH** (NCI YES; NIGMS BUILD, U-RISE); **NSF** (REU, LSAMP, S-STEM)
Pre-Doctoral	**NIH** (NRSA)

**Table 3.** Examples of various perspectives influencing demographic data considerations.

Perspective	Demographic Data Considerations and Use Cases

**STEM Trainee**	• “I’m in the category that gets left off of [federal data collection forms]. I want the longer survey. It’s worth my time to have me at the end of the list.” • [M]y lens is very much supporting students with disabilities in biomedical research training programs. And so for me, gathering the data is so we can inform these programs that these people are here, so the programs can be universally designed, hopefully. [...P]eople with multiple diagnoses are really, really underrepresented. [...] Some people disclose, some people don’t [...]. I know they’re there, but I feel like the programs don’t know, so that’s why it’s really important to me.”
**Program Staff with Direct Student Support**	• “I work with a lot of students - Middle Eastern students - who struggle a lot with the fact that their racial category is officially White [in U.S. Census]. But if you’re a Syrian refugee, and you’re trying to write an application [for scholarships or graduate school], and they ask you questions about underrepresentation, how do you navigate this?” • “I’m very aware we’re serving people that are dealing with the reality that those [demographic] questions may be dangerous to them. So, I’m thinking about end users.” • “When we are measuring NIH underrepresentation using the multi-step process, many students are underreporting their “underrepresentation” according to NIH categories [i.e., first-generation college status, rural geography], so they are underestimating their eligibility for scholarships.”
**Program Administrators**	• “We get to work with many culturally-focused programs and organizations. I find that their educators and education directors are justifiably concerned about asking questions of their youth and families that may feel invasive or that it may be used in a way that doesn’t benefit them. One of my hopes is that there is an opportunity for youth and families to answer questions that make them feel proud, and be seen.” • “Those same programs often have to respond to [demographic] questions in grant applications from organizations of privilege. They envision a room full of rich White people that are making judgments about their diverse populations. I just watch the frustration of folks who are having to respond to these [diversity] questions when they’re doing such an amazing job [and represent] the youth that we’re expanding STEM access to serve.”
**School Administrators**	• “There are different things that we do in different contexts. So when we’re looking at the [state data in order to know] ‘is there a pattern to the replication of privilege in [STEM field] education?’ Well, we know the answer is, yes. But if we start asking ‘are these interventions of this curriculum vs. that curriculum, or this professional development vs. that professional development, helping to disrupt those patterns?’ That’s a question we want to ask respectfully, and we want to use the answer to guide us forward. So that’s something I want to see how to do. [...T]here’s an authenticity to [...] individual stories that’s incredibly powerful, but when I’m looking at [state data], that’s not available. So, I’m trying to map that stack of problems [...] realizing that [aggregated demographic subgroups] are not robustly represented and there’s intersectionality with refugee and immigration status, and I’m not unpacking that when I go ‘ah, [this demographic group] aren’t a problem.’ That’s just disrespectful wrong thinking that distract[s] from an issue. I want to see, how can I use interaction with this community to avoid those pitfalls?”
**STEM Evaluators**	• “From my perspective, what I hear from programs is that they’re wanting guidance on how to operationalize these variables and what they should keep in mind so [data collection doesn’t] go errantly wrong. [...T]hat way others aren’t going to be potentially falling in similar pitfalls, or they know that they’re not alone in falling into those pitfalls as we work to figure it out.” • “Figuring out where data needs to be comparable and where it doesn’t need to be comparable - where demographic data is specific to a study where it needs to be comparable to another study in order to get broader data. And then we’re talking about if data needs to be comparable across studies or across categories. [If we’re supposed to be] having the same categories [...]; what are the categories everyone should be using? [...] Who is everyone? [...w]hen people try to make a single thing that is used for everyone, sometimes the response is to make it very long and unwieldy.”
**Equity-Focused Researchers**	• [Diversity] should inform scholarship and policy at the school [to] explicitly challenge recognized categories of marginalization. There are, for example, Indian tribes and nations that are recognized federally and not recognized federally [same for at the state and Tribal levels]. And to say, we are only going to start with the ones that are already recognized is to participate in the colonization and exclusion of already marginalized groups.” • “This topic gets thorny. For example in the context of parents or other caregivers for minors, sexual orientation minority or gender minority minors may or may not be ‘out’ to their caregivers, who may be supportive, non-supportive, or actively hostile. There are likewise intersections with legal systems in varying contexts where minors are or are not guaranteed confidentiality of their demographics from their parents/caregivers, and varying contexts where parents/caregivers are or are not guaranteed access to their minors’ demographic information.”

**Table 4.** Warnings and potential pitfalls in the pursuance of inclusive demographic practices.

Warning	Description and Rationale of the Warning or Concern

**Stigma and Harm Can Be Associated with Demographic Data**	• **Above all, do no harm to students**. For many people, facing something on a demographic check-off is highly stressful. Some data can cause exclusion or high levels of stigma, which can make an extremely severe practical difference if you disclose it, particularly in some geopolitical settings (e.g., LGBTQIA+). It may not be safe to disclose some demographic data or diagnoses, with some topics rarely feeling safe to identify (e.g., mental health/cognitive impairment). Disclosure has practical implications, as reporting for physicians can affect licensing in some states (Wible and Palermini, 2019) —there are real-world consequences of disclosure. • **Disclosure and non-disclosure are choices that people make for their own safety,** particularly for queer and trans people. “By failing to ask questions about people’s experiences that are accurate or offering opportunities for them to disclose, we are enforcing a ‘closet’” (i.e., a masking that hides authentic identity).
**Demographic Disclosure Should Be an Option, Not a Requirement**	• **The “why” is needed first.** Being asked to disclose in a vacuum is not going to yield accurate data unless the people know why they’re disclosing and what the practical result of that sharing would be (see Table 6). • **Offering students the option to disclose demographics supports the ownership of their personal narrative; but forcing disclosure puts students’ autonomy and sovereignty at risk.** “I have the right to decide whether to disclose something about myself, and if you don’t offer me the opportunity to do so, particularly something that is relevant to my experience here in STEM, as an underrepresented person, if I don’t have the opportunity to disclose, that is stripping me of some of my freedom to be wholly myself in a space.”
**Confront Who Has Decisional Power**	• **Who holds the power to decide?** Inequities can be perpetuated by individuals thinking they are doing the right thing. Be mindful of who holds the power versus who should have the power to decide demographic practices. Be mindful to not define data practices solely by a funder since there is NOT one standard frame (Appendix A). • **Power dynamics influence data collection approaches**. If using established categories, look for populations being marginalized: “to start [only] with [Tribes or other populations that] are already [federally or state] recognized is to participate in the colonization and exclusion of already marginalized groups.” Understand that “‘recognition’ directly refers to a power dynamic; one side is doing the recognizing.” Instead, look for how you can include the authentic identities of students in your programs in data collection.
**Excluded Groups**	• **Data disaggregation can help students feel seen**. While there is never one exhaustive list of demographics, data disaggregation can serve as a starting place. REALD (McGee, 2020) is one example (see Appendix C). • **Know your audience**. It influences the language needed to ensure understanding (e.g., English as a second language) or which groups should be added to make your students feel seen (e.g., immigrants; refugees; sex minority, sexual (orientation) minority, and gender minority (SSGM); individuals from specific Pacific islands or regional groups; Tribal affiliations, etc.). Know that checkboxes will always exclude someone, so be sure to include open-ended prompts that enable self-identification (see Table 6). • **Disability is highly nuanced and intersectional.** REALD (McGee, 2020) offers a tool for capturing functional limitations associated with disability, which can be used to improve accessibility. Disability can extend beyond medical diagnoses; consider social connections among individuals with disabilities and neurodivergence who may want to connect with others like them for their own empowerment and strengths-based support.
**Avoid Assumptions**	• **Underrepresented populations can be more prevalent than expected.** While some demographic groups may be perceived as a small minority (e.g., non-binary), this is not the case across all populations and cultures (Hunt, 2016; Joel et al., 2013; Monro, 2019). • **At-birth assignments are not universal**. Not all individuals are assigned sex-at-birth or assigned a binary gender at birth (Holzer, 2019; Lamm, 2019; US Birth Certificates, n.d.) and more families are raising children in gender-neutral environments (Matei, 2020). • **Be wary of stereotyping**; recognize that any population will have a distribution of beliefs, identities, diversities, or behaviors. Intersectionality is often missing; look for it.

**Table 5.** Uses for demographic data that improve STEM training and supports for students.

Use	Description and Rationale of Use

**Invite and Welcome Individuals; Their Representation is an Important Part of the Group**	• **Recognizing and validating social identities makes people feel more welcomed,** more invited, and more willing to participate. Self-Determination Theory (Ryan and Deci, 2020) describes relatedness as important for supporting a student’s sense of belonging in STEM. Inclusive demographics can make students feel seen, welcomed, and that they are not alone. • **Improve visibility and communication across individual perspectives.** Discussion of peer representation can help trainees better understand perspectives within their learning community. Learning “with, from, and about each other” is essential for interprofessional education (Health Professions Accreditors Collaborative, 2019). Provide ways for students to share aspects of their identities in safe ways that do not “out” them.
**Provide Tailored Support**	• **Provide mentors that match the backgrounds or experiences of students.** “In our graduate school peer mentorship program, individuals identified the demographic, identity, or experiences that they wanted mentorship on as first year graduate students. They ranked those in the order they wanted mentorship and we matched based on their order to best meet their needs. We didn’t limit it to demographics of mentors and mentees. [Rankings were used to] actually [match] people on what they wanted mentoring around as they came into graduate school, instead of making assumptions, because they fell into a particular category of [underrepresentation]. [It’s about] where they wanted the mentorship.” • **Incorporate trauma-informed practices.** If a STEM program is offering writing support with [college, scholarship, graduate] applications, recognize that ‘the diversity question’ on applications can often be very challenging for students who represent underrepresented or marginalized backgrounds. It can resurface past trauma, discrimination, or oppression. STEM programs may not have experience in supporting these students, so recommending that students disclose these traumas in the service of promoting diversity can be very harmful to students and their application. STEM programs should be mindful of how they ask this question and how to counsel trainees to respond to it in essays.
**Direct Individuals to Group-Specific Resources**	• **Improve access to resources.** Consider what resources may best serve students representing a particular demographic group. Just as people who get pregnant may need pregnancy-specific health care, students of demographic groups may benefit from additional resources, such as cultural or identity affinity group support, disability resource centers, or financial aid support. Some students may be more eligible for more scholarships than they previously understood. • **Recognize intersectionality.** First-generation college students may be unfamiliar with the complexity of financial aid (e.g., FAFSA); academic advising can help guide decisions so students don’t exhaust financial aid during their coursework. Demographic groups may have specific financial needs (i.e., students of Islamic faith who practice ‘riba’ will not take out loans that accrue interest, so their financial support may be limited to scholarships, stipends, and incentive programs (Marriott et al., 2021)).
**Programmatic Feedback for Process Improvement**	• **Incorporate feedback from target audience to understand where growth can occur.** Look for how feedback from students can be solicited to improve how to serve target populations and ensure accessibility. • **Trainee advisory boards can provide specific guidance.** Similar to teacher advisory boards or cultural advisory boards, recognize that trainee advisory boards can help STEM programs think through their program activities, demographic data practices, and advise on approaches for recruiting and retaining a diversity of STEM trainees. Program alumni are often great resources and advisory board participation can be described on their resumes/CVs to demonstrate their professional experience. • **Near-peer mentors can improve feedback loops to programs** while supporting students in culturally-specific ways (Huerta et al., 2022).

**Table 6.** Recommendations for establishing demographic data practices and procedures.

Practice	Description and Rationale of the Practice

**Establish Transparency Around Data Policies and Procedures Needed for Safeguarding Student Privacy**	• **Provide the why.** Being asked in a vacuum to disclose is not going to yield accurate data unless people know why they’re disclosing and what the practical result of that would be. For example, “are you going to match demographic mentorship? Are you going to give additional services? Why are you asking? How are you going to use it? What’s the benefit for students moving forward, rather than just giving stuff away?” • **Describe the safeguards.** Answer core questions in advance like “what are you going to do with the data?” and “who will have access to my information?” These questions are essential for data sovereignty and student autonomy. Individuals representing certain identities may have strong concerns about “being on a list somewhere.” Building trust is a foundational practice for STEM training and research. Programs should aim to become trustworthy.
**Consider the Audience and Timing of Demographic Data Collection**	• **Timing is important.** Even when “why” is provided, recognize that asking demographic questions at the onset (i.e., time of application to a program) can be uncomfortable and that not all data have the same amount of sensitivity/concern. Programs do not have to ask all demographic questions at one time. Disaggregated data collection takes time for participants to complete, so the implementation setting (e.g., an informal STEM program) matters as does the privacy participants are given to answer sensitive questions. • **Audience age will influence demographic questions and clarification needed.** Younger audiences may be unfamiliar with what demographic questions are asking (i.e., ethnicity, disadvantaged background, or sexual orientation identity, etc.; Nuances in Appendix C). • **Allow for shifting identities.** Avoid the assumption that demographics don’t change. Instead, recognize that asking for demographics over time enables students to disclose identities based on their comfort with your STEM program or changes in life experience. This may especially be the case with disability and sexual orientation/gender identity (SOGI).
**Don’t Conflate Data Collection with Reporting**	• **Data collection should make participants feel welcome.** While programs may use checkboxes to make data collection and reporting easier, particularly for larger programs and studies, recognize that not all data have the same amount of sensitivity/concern. • **Open-ended prompts have value**. Instead of “other” on data collection forms, consider if an open-ended prompt may be more supportive of what you’re trying to accomplish. Individuals can self-identify (see Appendix C for phrasing from Marr, 2021). • **Make room for identities**. The question of “what are the demographic categories we should be interfacing and collecting?” is different from “what are the categories we should be analyzing and reporting?” Recognize that demographic identities (raw data) can be combined for analysis, reporting, and visualization of program impact. As reporting aims to protect student privacy, small sample sizes are often collapsed. Evaluators should mind distinctions, so students can feel seen while providing STEM programs with meaningful data.
**Make Room for Intersectionality; It is Rare for Trainees to Be One Demographic Category**	• **Multiple identities exist.** Recognize that students are not one demographic group; their experiences across demographic categories are intersectional. Students may have faced additional biases or discrimination based on these multiple identities; do not diminish these experiences. Instead, look for ways to support reporting of intersectional identities. • **Federal reporting of demographic summaries is insufficient.** Lumping intersectional data into a single category hides identity. Reporting racial/ethnic demographics on federal forms often does not permit nuanced identities, as the category “more than one race” may improperly aggregate data, leading to the underreporting of individuals from underrepresented racial demographic categories (see Appendix C). • **Language can shift based on demographic identity.** Individuals identifying as American Indian/Alaskan Native may use the term “Two-Spirit” to refer to non-binary identities, which is a long-standing term in their culture (Hunt, 2016). Be inclusive of these terms and identities.
**Improve Data Accuracy by Supporting Student Understanding, Privacy, and Safety**	• **Safety impacts disclosure**. Supporting the “why” of demographic data collection encourages accuracy, but does not guarantee it. • **Balance self-identification with awareness**. While self-identification is encouraged, evaluators should be mindful that populations are not only identity-based, but also exist through social and ecological positions. For example, while many U.S. residents identify as middle class, the material reality may differ for families making $30,000 vs $130,000 per year. Students may be unaware of the financial reality when answering questions. • **Asking follow-up questions can support verification and accuracy.** Instead of a single question about first-generation college status, consider also asking about the educational attainment of guardians. In prior work, students conflated associate’s degrees and older siblings pursuing bachelor’s degrees as not being first-generation college (Marriott, Raz Link, et al., 2022). Likewise, asking for zip code can help verify rural geography and health-professional shortage area eligibility for NIH disadvantaged background. Students who move frequently, due to homelessness or family military experience have more challenges. Open-ended prompts can enhance clarity and data accuracy if student confusion exists.

**Table 7.** Recommendations for students, programs, and funders.

Students	Programs	Funders

• Recognize that identity disclosure should be voluntary; do not feel obligated to disclose. • Look for ways to report back to programs about data practices that could be improved. • Request mentors who represent personal or professional identities of interest. • Be kind to others; share perspectives and recognize that peers may have faced significant barriers in their lived experiences.	• Make sure demographic questions have options for “prefer not to answer” and open-ended prompts that give students a range of options for answering. • Offer ways for students to anonymously report issues – whether in demographic data practices or within training programs more broadly. Safe reporting without retribution is essential. • Offer multiple opportunities when demographic questions are asked that support building trust and measurement of potentially changing identities. • Incorporate identity-focused mentorship for trainees. • Incorporate professional development in diversity, equity, inclusion, and accessibility for program staff; talk with external programs to learn about advancements.	• Expand federal recognition of marginalized underrepresented groups, such as SSGM and LGBTQIA+ individuals and those who have experienced significant displacement (Appendix A). • Provide guidance for STEM programs on phrasing for consistent demographic data collection and data practices for working with minoritized and emergent populations, ideally by establishing an evaluation center that can advise fundees. • Enable reporting of intersectionality in federal reporting forms to more fully describe trainee backgrounds and experiences. • Remove full-time program participation requirements for underrepresented trainees in STEM programs, particularly for students with disabilities and/or disadvantaged backgrounds.

## ABBREVIATIONS

CoSTEM: Committee on STEM Education
HPSA: Health Professional Shortage Area
HRSA: Health Resources and Services Administration
IDEA: Individuals with Disabilities Education Act
IEP: Individualized Education Program
LGBTQIA+: Lesbian, Gay, Bisexual, Transgender, Queer, Intersex, Asexual, etc
MENA: Middle Eastern and North African; NIH: National Institutes of Health
NSF: National Science Foundation
NSTC: National Science and Technology Council
REALD: Race, Ethnicity, Language, and Disability
SGM: Sexual and Gender Minorities
SOGI: Sexual Orientation and Gender Identity
SSGM: Sex Minority, Sexual Minority, and Gender Minority
START: STEM Assessment and Reporting Tracker
STEM: Science, Technology, Engineering, and Math
TGD: Transgender or Gender Diverse
US: United States
