# Supplementary material for: Inclusive Approaches for Measuring Demographics of Underrepresented Populations in STEM and Biomedical Research Training Programs
Source: J STEM Outreach. Author manuscript; Available in PMC 2024 Jul 12. (PMC11241867; doi:10.15695/jstem/v7i2.10)
Supplement: Parisetal2024_AppendixA_FedLandscape [file NIHMS2004214-supplement-Parisetal2024_AppendixA_FedLandscape.pdf]

JOURNAL of STEM OUTREACH
Vol. 7, Issue 2, February 2024

Inclusive Demographics in Biomedical Training – Paris, et al.

Appendix A. Federal Landscape of Underrepresented Populations

Table A1. *Demographic populations considered underrepresented by funders.*

**NIH**<br>Underrepresented populations in the U.S. biomedical, clinical, behavioral and social sciences research enterprise (NOT-OD-20-031; National Institutes of Health, 2019)	**NSF**<br>Underrepresented in science and engineering (S&E; National Center for Science & Engineering Statistics, 2023, page 5)	**Populations Marginalized or Excluded from Data Collection Efforts**
• Racial and Ethnic groups (Blacks or African Americans, Hispanics or Latinos, American Indians or Alaska Natives, Native Hawaiians and other Pacific Islanders<br>• Individuals with disabilities<br>• Individuals from disadvantaged backgrounds (comprising 7 variables, with 2 or more defining a disadvantaged background; homelessness experience, foster care experience, federal free and reduced lunch experience, first generation college student status, Pell eligibility, WIC eligibility, and rural geography or health professional shortage area)	• Racial and ethnic groups (Blacks or African Americans, Hispanics or Latinos, and American Indians or Alaska Natives<br>• Persons with disabilities<br>• Women	• LGBTQIA+ (Lesbian, gay, bisexual, transgender, queer, intersex, asexual and groups comprising sex minorities, sexual orientation minorities, and gender minorities; SSGM). Recommendations from White House (2023a) and defined in White House (2021a, b, d; 2022)<br>◦ Non-Western gender systems. For example, various North American Indigenous nations, tribes, and communities may use alternate terms, such as "Two-Spirit," which refers to non-binary identities and is a long-standing term in Indigenous culture (Hunt, 2016)<br>• Marginalization of specific racial and ethnic groups<br>◦ Middle Eastern/North African is not represented as a demographic option, with the U.S. census coding this group as "White"(Office of Management and Budget, 2019; Federal Register #58782, 1997<br>◦ Southeast Asian populations (e.g., Cambodian, Laotian, etc.) who are aggregated as Asian<br>• Marginalization or exclusion of experiences<br>◦ Immigrants and refugees<br>◦ Veterans<br>◦ Parents and caregivers, which impact ability of trainees to participate full-time in advanced schooling and STEM training programs<br>• Religious or cultural minorities<br>◦ Those who hold religious beliefs or cultural practices that are different from those of the majority or dominant culture have reported feeling isolated, excluded, discriminated, and professionally disadvantaged (Marks et al., 2019).

Table A2. *Executive Orders (EO) calling for equity in data collection practices.*

**Executive Order Number**	**Executive Orders for Equity in Demographic Data Collection**
**EO #13895**<br>(White House, 2021a)	Executive Order On Advancing Racial Equity and Support for Underserved Communities Through the Federal Government #13985: "Sec. 4. Identifying Methods to Assess Equity. (a) The Director of the Office of Management and Budget (OMB) shall, in partnership with the heads of agencies, study methods for assessing whether agency policies and actions create or exacerbate barriers to full and equal participation by all eligible individuals. The study should aim to identify the best methods, consistent with applicable law, to assist agencies in **assessing equity with respect to race, ethnicity, religion, income, geography, gender identity, sexual orientation, and disability**" (White House, 2021a).
**EO #113988**<br>(White House, 2021b)	"[T]o prevent and combat discrimination on the basis of **gender identity or sexual orientation**, and to fully enforce Title VII and other laws that prohibit discrimination on the basis of gender identity or sexual orientation. It is also the policy of my Administration to address overlapping forms of discrimination" (White House, 2021b).
**EO #14031**<br>(White House, 2021c)	Advancing Equity, Justice, and Opportunity for Asian Americans, Native Hawaiians, and Pacific Islanders (#14031): "(iv) To expand the collection and use of **disaggregated data** at the Federal, State and local level on AA and NHPI communities, and facilitate improved research on policy and program outcomes for AA and NHPI communities, in coordination with the Interagency Working Group on Equitable Data established by Executive Order 13985" (White House, 2021c) .
**EO #14035**<br>(White House, 2021d)	Requires federal agencies to **promote diversity, equity, inclusion, and accessibility** across the federal government by establishing an Equity Task Force. The order also mandates a "preliminary assessment of the current state of diversity, equity, inclusion, and accessibility in the agencies human resources practices and workforce composition" (White House, 2021d).
**EO# 14075**<br>(White House, 2022)	Executive Order on Advancing Equality for Lesbian, Gay, Bisexual, Transgender, Queer, and Intersex Individuals: "Sec. 11. Promoting Inclusive and Responsible Federal Data Collection Practices. (a) Advancing equity and full inclusion for LGBTQI+ individuals requires that the Federal Government use evidence and **data to measure and address the disparities that LGBTQI+ individuals**, families, and households face, while **safeguarding privacy, security, and civil rights**." (White House, 2022; 6/15/2022). Recommendations for language have begun to be addressed (White House, 2022, 2023a).
**EO# #14091**<br>(White House, 2023b)	Executive Order on Further Advancing Racial Equity and Support for Underserved Communities Through The Federal Government. This order builds upon previous equity-related Executive Orders by extending and strengthening equity-advancing requirements for agencies by **establishing and/or advancing equity-focused leadership, processes, policies, procurement, data practices, and partnership with underserved communities**; creating economic opportunity in rural America and advancing urban equitable development; and affirmatively advancing civil rights (White House, 2023b).
