# Supplementary material for: Inclusive Approaches for Measuring Demographics of Underrepresented Populations in STEM and Biomedical Research Training Programs
Source: J STEM Outreach. Author manuscript; Available in PMC 2024 Jul 12. (PMC11241867; doi:10.15695/jstem/v7i2.10)
Supplement: Parisetal2024_AppendixB_Positionality [file NIHMS2004214-supplement-Parisetal2024_AppendixB_Positionality.pdf]

Inclusive Demographics in Biomedical Training – Paris, et al.

Appendix B. Positionality Statements of the Co-Author Team

Positionality statements that critically examine and represent researchers' identities in relation to existing power dynamics in their research contexts allows readers to gain insights into how co-authors' personal perspectives may influence their research choices, including opportunity to disrupt privilege within their own work (Secules et al., 2021). The positionalities of the co-author team are summarized in Table B1.

Table B1. *Summary of co-author team positions.*

Co-Author	STEM Trainee	STEM Education Programs	Evaluator	Equity Researcher*	STEM Faculty & Associate Deans	Historically Underrepresented in STEM?^
Abel, Jerian		X (K-12)	X			Yes (NSF)
Alvord, Teala W.	X		X	Secondary		Yes (NIH)
Chase, Kieran	X			Primary focus		Yes (NIH)
Dinno, Alexis				Primary focus	X, Public Health	Yes (WH)
Espinosa, Shanthia	X	X (HS, undergraduate)		Secondary		Yes (NIH)
Harrison, Kristen		X (P-20)				Yes (NSF)
Hook, James					X (Assoc. Dean)	No (NSF)
Lentz, Brandy	X	X (undergraduate)		Primary focus		Yes (NIH)
Marr, Mollie C.	X			Primary focus		Yes (NIH)
Marriott, Lisa K.		X (MS-graduate)	X	Secondary	X, Public Health	No (NIH)
Paris, Stephanie E.	X			Secondary		Yes (NIH)
Raz Link, Aaron		X (undergraduate)				Yes (NIH)
Richardson, Dawn M.				Primary focus	X (Assoc. Dean)	Yes (NIH)
Setthavongsack, Angie	X	X (undergraduate)				Yes (NIH)
Shusterman, Gwen					X, Chemistry	Yes (NSF)

STEM education programs include student-serving staff and support of individuals, programs, and organizations at the P-20 level to improve access and quality of STEM education.
**Equity researcher denotes individuals whose primary focus is equity research and are experts in the field. Co-authors who specialize in other areas, but often work in the realm of equity research are denoted as secondary.*
^Underrepresentation in STEM is defined by each co-author based on the funding agency with whom they primarily work (NIH or NSF) or based on White House equity definitions (WH) that are more inclusive "with respect to race, ethnicity, religion, income, geography, gender identity, sexual orientation, and disability" (White House 2021a; Appendix A).

In alphabetical order by last name:

Jerian Abel, PhD (she/they) is co-director of the Portland Metro STEM Partnership (PMSP), responsible for the research and evaluation as well as training and technical assistance related to evaluation, assessment, and program design. Duties shared with the other co-director support the Collective Impact initiative, including planning and facilitating events and supporting various teams and workgroups. In addition, she manages a portfolio of projects that include K-8 STEM Schools Support, Joy & Justice in Math K-8 professional development, Design Thinking for STEM Equity, among others. Prior to joining PMSP, Dr. Abel worked for 16 years at Education Northwest, a regional education laboratory, holding a variety of management and research positions on federal, state, and district grants and contracts. Projects ranged from providing training and technical assistance to nonprofits and grantmaking institutions implementing Collective Impact, developing online courses & facilitating online Communities of Practice; conducting research and evaluation on school reform; and working directly with schools and districts to support school improvement processes. Prior to Education Northwest, Dr. Abel worked with K-12 educators to improve science education in rural Southwest Virginia. Her teaching experience spans high school (physics & math) through graduate studies and includes work with both preservice and inservice teachers. Prior to entering the education field, Dr. Abel worked as a practicing research scientist on a variety of applied Research & Development projects funded through the National Institutes of Health, Department of Energy, and Department of Defense. She has a Ph.D. in Curriculum and Instruction with an emphasis on Instruction Systems Design and a Master of Science in Interdisciplinary Science and Science Education. Growing up Queer in rural North Carolina and Southwest Virginia, she experienced and witnessed too much hate and discrimination against LGBTQIA+ individuals. At the same time, she had limited exposure to positive Queer role models along the continuum of LGBTIA+, an area of interest that remains to this day.

Teala W. Alvord, MPH CPH (she/her) is an evaluation coordinator with the Clark County Washington Public Health Health Assessment and Evaluation team, a graduate of the OHSU-PSU School of Public Health, a former research assistant and lab manager with the Marriott Lab, and a NIH-funded BUILD EXITO alumna. Teala grew up in a low-income home headed by a single mom in San Francisco, and was a PELL grant recipient through her undergraduate years. Teala is also proudly recovering from substance use disorder in her late teen years, and is passionate about working to reduce stigma surrounding mental and emotional health, substance misuse and abuse, and harm reduction approaches. As an evaluation coordinator for Clark County, Teala supports community partners with technical assistance in assessment and evaluation projects focused on racial, gender, income, and other types of equity, is conducting a qualitative evaluation of a harm reduction program focused on opioid use disorder in SW Washington, and leads qualitative research method trainings for community partners working with youth to prevent violence and substance misuse and abuse. As a graduate research assistant, Teala supported the evaluation of a food access program serving low-income and historically excluded and marginalized populations accessing care at many of Portland's Federally Qualified Health Centers. And, as an undergraduate researcher in the BUILD EXITO program, Teala supported Dr. Marriott's work incorporating more inclusive demographics to better represent individuals participating in research and evaluation, developing protocols designed to address and mitigate disparities for excluded and marginalized students, and mentor and train youth from diverse, underserved populations across Oregon. Teala benefits from several intersecting privileges, being born white-skinned, able-bodied, heterosexual, and cis-gendered, As such, she continues to reflect on the ways in which she continues to participate in upholding oppressive structures of power, and how she can work to cede that power and aid in redistribution of it, as a central tenet of her work and life.

Kieran Chase, BA (they/them) is pursuing a Master's of Public Health focused on epidemiology. Chase is a first-generation college student from a rural, medically underserved community in the midwest. They grew up a recipient of federal free lunches and other forms of government assistance, and participate in academic institutions as a person with multiple learning disabilities. Their professional experience prior to pursuit of this degree has focused on community and campaign organizing, policy advocacy, administrative equity policy writing, and workplace equity training targeting topics of sex, sexual orientation, gender identity, and gender expression. As a trans person who has been engaged in both advocacy and scholarship related to advancing the wellbeing of the trans population, Kieran is committed to community-engaged processes to effect change, whether via research or policymaking. Kieran's primary research interest is in social epidemiology; that is, in understanding how social forces and macro-social structures shape individual and population health. Kieran has previously worked as a research assistant with Dr. Alexis Dinno at the OHSU-PSU School of Public Health on projects related to accurate and inclusive demography and demographic data collection for identities and social positions related to sex, gender, and sexuality. They currently work as a teaching assistant supporting student learning in introductory biostatistics courses at the same institution.

Alexis Dinno, ScD, MPH, MEM (she/her) is an Associate Professor in the OHSU-PSU School of Public Health where she teaches graduate courses in quantitative methods, health equity, and social theory. She has experienced privileges and adversities. Some of the life experiences informing her work in the sciences have included childhood poverty, feminism, domestic violence, parental drug use, cisgender & transgender, transsexual, bisexual, lesbian, immigrant family, multi-ethnic Iraqi American, food stamps, mental illness, anti-war movement, capoeirista, musician, dancer. She has collaborated with the Oregon AIDS Education & Training Center, the Oregon Department of Human Services, and the Oregon Health Authority in creating, refining, implementing, and educating around inclusive and equitable demographic standards including REALD (Race, Ethnicity, Language & Disability) and SOGI (Sexual Orientation & Gender Identity), including novel work with SOGI and minors. She has also worked with the American Thrombosis and Hemostasis Network, and the National Hemophilia Foundation (NHF) both as an expert in inclusive SOGI demographics, and as a diversity, equity and inclusion collaborator in strategic planning for the NHF's long term research program. She served for seven years in the OHSU-PSU SPH's diversity, equity and inclusion committee, including two years as chair, where she was a primary author on institutional definitions of 'diversity' and 'equity', and where she was the primary developer and promoter of "A Reflexive Tool for Building Equity & Inclusion Into SPH Courses". She has recently assisted the Prevention Research Center of the Pacific Institute for Research and Evaluation apply for Health Equity Research Hub funding from the National Institute of Health's ComPASS Program in the role of a Multidisciplinary Expert Panel member specializing in biostatistics and

social epidemiology. She publishes and routinely provides support for several statistical software packages, and is a regular contributor to CrossValidated, the statistics-themed curated question & answer website in the StackExchange network, where she is ranked in the top 0.5% of global contributors.

Shanthia Espinosa, BS (she/her) is a Senior Research Assistant for the Marriott lab at the OHSU-PSU School of Public Health. She brings a unique perspective as a first-generation Chamorro (Pacific Islander) Dominican-Haitian woman from Saipan, a small island in the Commonwealth of the Northern Mariana Islands (CNMI) where opportunities to be in STEM are finite. As a marginalized, low socioeconomic status, Pacific Islander woman of color in STEM, Shanthia takes pride in being a recipient of the NIH-funded BUILD EXITO scholarship, an undergraduate biomedical research program that supports first-generation, underrepresented and underserved communities in STEM. In her dedicated role, Shanthia serves as a community advocate for biomedical research programs, providing valuable guidance to students of color through research, mentorship, outreach, and community engagement. As an undergraduate research trainee in the Marriott lab, her research explored STEM motivation supports for Pacific Islander undergraduates. Continuing her passion for motivational research in STEM and in Pasifika communities, Shanthia conducted research with faculty mentor Dr. Alma Trinidad as a Ronald E. McNair scholar on “Motivational Factors and Opportunities in Science, Technology, Engineering, and Mathematics (STEM) fields among Native Hawaiian Pacific Islanders (NHPI),” an ongoing paper in progress to be submitted for publication. As a Senior Research Assistant, Shanthia is passionate about disaggregating data through inclusive data practices. Her research interests align with her long-term goals of highlighting the importance of inclusive demographics in training programs to inform diverse outcomes for students’ success through equitable and accessible mentorship. She plans to pursue an MPH in Public Health Practice and conduct research supporting marginalized communities in educational and STEM pursuits.

Kristen M. K. Harrison, MTE (she/her) is co-director of the Portland Metro STEM Partnership (PMSP) responsible for partnership development and engagement. Duties shared with the other co-director support the Collective Impact initiative, including planning and facilitating events and supporting various teams and workgroups. In addition, she manages a portfolio of projects that include STEM in Early Learning, Elementary STEAM Leaders, the Collaboratory (an initiative focused on supporting out of school STE(A)M programs), the BIPOC STEAM Ecosystem (an initiative of and led by eight culturally-focused programs and organizations), STEM Beyond School, High School Science for All, Mathways, and Computer Science for All. The work with the Collaboratory and BIPOC STEAM Ecosystem partners was a driver for participation in this article. Many of the Collaboratory partners and all of the BIPOC STEAM Ecosystem partners are culturally-based and designed to engage and empower diverse youth. Many partners are part of both the Collaboratory and BIPOC STEAM Ecosystem. Previously, Ms. Harrison worked for Ignite Ed, formerly Industry Initiatives for Science and Math Education (IISME) where she developed professional development and coached educators on best practices in STEM education including hands-on, minds-on, and real-world relevant learning. Ms. Harrison was also a high school science and math teacher. Her experience teaching Conceptual Physics to students deemed not “ready” for Chemistry showed how our pathways in education harm students, their identities, and their futures. Ms. Harrison holds a Bachelor of Science in Natural Sciences with a focus in Biology. She also holds a Masters in Teacher Education.

James Hook, PhD (he/him) is a Professor of Computer Science and Associate Dean of the Maseeh College of Engineering and Computer Science at Portland State University. Dr. Hook experienced education through a lens of privilege, and with little recognition of the implications of that privilege. His engagement with issues of equity in education grew out of a sense of urgency that radical change was necessary to disrupt the patterns of exclusion that made computer science a highly segregated discipline by gender and race. Awareness and good intentions failed to reverse patterns of exclusion in computing. As Associate Dean at Portland State University (PSU), he focuses on increasing the representation of historically excluded identities in the graduating class. His efforts have expanded beyond higher education to include building capacity for inclusive education throughout the educational ecosystem. As the PSU sponsor of Oregon MESA and the Portland Metro STEM Partnership (an Oregon state STEM Hub co-directed by Dr. Abel and Ms. Harrison) he has engaged equity-focused initiatives that address P-12. He is principal investigator (PI) of a Computer Science (CS) program (CS for Oregon), a collaborative project with education and computer science faculty at the University of Oregon and Oregon State University-Cascades based on building inclusive CS education capacity in Oregon high schools. He participates in the Expanding Computing Education

Pathways (ECEP) alliance as a member of the Oregon team. ECEP is an National Science Foundation (NSF)-sponsored national broadening participation in computing (BPC) alliance; the Oregon team includes representatives from the Oregon Department of Education. He oversees the PSU Louis Stokes Alliance for Minority Participation (LSAMP) program. LSAMP is an NSF program to support underrepresented students in STEM disciplines. He is the STEM Administrator for the EAGLES S-STEM project, an NSF-sponsored needs-based scholarship program awarded to PSU and Heritage University, a tribal college of the Yakima Nation. He has recently worked with Professor Shusterman on the Student Experience Project, a multi-institution promoting the adoption of evidence-based practice for inclusive pedagogy in gateway STEM courses led by the Urban Serving University coalition of the Association of American Public and Land-grant Universities.

Brandy L. Lentz, BS (she/her) is a first year MPH student in the Health Management and Policy program at OHSU-PSU School of Public Health. She plans to continue her training to make healthcare and education more accessible. She has been a researcher with a focus on health and education equity for underrepresented STEM students for the last five years. She is an experienced mentor who has provided support for countless undergraduate students coming from underrepresented communities as they pursue STEM education. She is a fierce advocate for disabled students and a proponent of the application of Universal Design Principles to research and education. As a Queer, disabled, woman in science, she has valuable lived experience that informs her work and provides insight into ways to support underrepresented communities that may otherwise go unnoticed. As a NIH-funded BUILD EXITO scholar, she has served as a research assistant in multiple labs during her undergraduate and post-baccalaureate training. She applies concepts and theories to real world research to support accessibility within biomedical research training. She is passionate about neurodiversity and wants to continue to do research within health systems to serve people with disabilities. Her prior work applied qualitative methods to explore the experience of burnout for autistic adults. Her skills and knowledge are relevant to the service of developmentally disabled patients, including those with attention deficit hyperactivity disorder (ADHD) and autism spectrum disorders. She has been working with a collaborative team to understand strength-based supports for impulsivity in the context of STEM engagement, which uses health informatics for data collection and returns personalized e-feedback.

Mollie C. Marr, PhD (she/her) is a first year resident in the Massachusetts General Hospital (MGH)/McLean Adult Psychiatry Residency Program. She is a recent graduate of the Medical Scientist Training Program at Oregon Health & Science University. She received an NIH F30 grant from the National Institute of Mental Health (NIMH) to study the intergenerational transmission of childhood maltreatment. She is a current trainee from a disadvantaged and low-income background who has developed programs to support students interested in science and medicine. She is an LGBTQ+ scientist who is passionate about the representation of low-income and LGBTQ+ individuals in medicine and science. Currently, she is engaged in work to address racial and gender bias in letters of recommendation and evaluation (https://github.com/gender-bias).

Lisa K. Marriott, PhD (she/her) is an Associate Professor in the OHSU-PSU School of Public Health studying applied science education and biomedical workforce development. While not historically underrepresented in STEM herself, she frequently works with student-serving STEM programs. Lisa experienced privilege in affording college and STEM experiences during her youth, privileges that are not universally experienced by the trainees with whom she works. Lisa is passionate about accessible STEM education and training for all. She has served as principal investigator of three NIH-funded Science Education Partnership Awards (SEPAs) focused on data science for K-12 students. Resources developed through her SEPAs support international research with ongoing projects in Thailand. She is co-investigator of cancer-focused Youth Enjoy Science (NCI YES) training programs for high school and undergraduates, and co-investigator of a NIGMS-funded BUILD program working with undergraduate trainees. Her SEPA grant built the STEM Assessment and Reporting Tracker (START) to help STEM programs measure the STEM development of their students, in which demographics are a core function. She used to serve as co-director of OHSU's Evaluation Core and is currently the principal investigator of an Institutional Review Board (IRB)-approved study on Biomedical Workforce Development (OHSU IRB #22889). She works with near-peer mentoring teams across STEM training programs and works with Dr. Marr on her IRB-approved studies investigating women and LGBTQIA+ scientists in the biomedical workforce. Together, these STEM programs and research studies have helped her grow significantly in learning about demographics and their intersections with student experiences that greatly shape STEM trajectories. She was awarded a NIH award for Excellence in Diversity, Equity, Inclusion, and

Accessibility Mentoring in 2022. Her ongoing work aims to improve the STEM training landscape for current and future trainees.

Stephanie E. Paris, BS (she/her) is a Research Project Coordinator and Lab Manager for the Marriott lab at the OHSU-PSU School of Public Health. She is also a graduate student in the Master of Professional and Technical Writing program at Portland State University, which aligns with her long term research goal to use a variety of dissemination methods for health promotion. One of her intrinsic values is being of service to others, and her work in the Marriott lab supports the fulfillment of this purpose by offering opportunities to contribute to the innovation and dissemination of protocols that mitigate disparities and health inequities in marginalized and institutionally underserved populations. Stephanie has learned firsthand how expanded, inclusive demographics can support intersectionality in research. Although she does experience many privileges because she is white, cisgender, and straight, and both her parents went to college, she is also categorized as disadvantaged and underrepresented in her field because she has cognitive deficits that can often be disabling, low socioeconomic status, is a woman in STEM, and grew up in a qualifying Health Professional Shortage Area. Many of these disadvantages contributed to multiple failed college attempts over the course of about 20 years, until she found the NIH-funded BUILD EXITO research training program, whose supports for students from a diversity of backgrounds allowed her to finally obtain a college degree and begin her career in research. As someone who suffers from a non-visible neurodevelopmental disorder, one dilemma Stephanie understands is the difficult choice whether to disclose her functional limitations in order to gain access to services and accommodations that would increase her potential for success, or continue attempts to mask her difficulties to avoid the stigma attached to being viewed as disabled; the latter has a unique way of negatively impacting health and well-being, and exacerbating disabilities. Ultimately Stephanie believes that with appropriate support and accommodations, individuals with disabilities and neurodivergence can achieve successful careers and lead meaningful and fulfilling lives.

Aaron Raz Link, MFA, MA (he/him) works with the NIH-funded BUILD EXITO program as an instructional lead and manager of the Scholar Enrichment program, which serves underrepresented students and alumni of multiple research training programs (i.e., BUILD EXITO, URISE, DREAM) within the Center for Interdisciplinary Mentoring Research at Portland State University. His areas of research interest include hidden and implicit curriculum, stigma, and self-representation. He is a two-time Regional Arts and Culture Council (RACC) grantee for community-based projects on identity, and a Lambda Literary Award finalist for nonfiction writing. A primary area of practice is teaching people from diverse marginalized populations to craft language for professional identity and application documents that will both accurately represent their backgrounds, and give them access to funding and professional credentials in U.S. academic and medical systems. His qualifications include twenty years of teaching and mentoring experience in settings including academic degree programs, major public science museums, and a homeless service agency. He co-created the inspiration2publication program of Antioch University Los Angeles, has graduate degrees in Nonfiction Writing and the History and Philosophy of Science, and survival experience as a disabled Jewish gay trans man from Nebraska who transitioned in the early 1990s. Two primary influences on his perspective on gathering demographic data on individuals are generational: 1) Like many American Jews, he grew up around refugees from the Holocaust, the Islamic Revolution in Iran, and the Cambodian genocide. 2) Like many trans people, he has been undocumented, with an identity demographic that officially did not exist, in a legal status that included no human rights.

Dawn M. Richardson, DrPH, MPH (she/her) is the Associate Dean for Social Justice and the Interim Associate Dean for Academic Affairs in the OHSU-PSU School of Public Health (SPH), where she is also an Associate Professor of Health Promotion and Community Health. A social epidemiologist, Dr. Richardson focuses her research questions on the pathways by which the unequal distributions of income, power and wealth (shaped and reified by structural racism) affect social and geographic mobility, access to opportunity, and ultimately health outcomes for women of color. Working in partnership with communities impacted by inequities, she incorporates research findings into concrete programs and policies to promote population health. Her most recent research efforts include an NIH-funded study examining the role of documentation status on Latina immigrant women's health; an evaluation of paid leave policies and related analysis of structural racism as a driver of barriers to accessing such policies; and a pilot project aimed at understanding how to best support BIPOC Women Scholars pursuing STEM-focused degrees to promote public health. Her scholarship is rooted in her lived experience as a Mexican American, queer, first-

generation scholar from East Tennessee.

Angie Setthavongsack, BS (he/she/they) is a first generation Laotian-American. They plan on applying to an MPH program in Global Health. During undergrad, they were heavily involved with Diversity & Multicultural Student Services (DMSS) and Student Activities and Leadership Programs (SALP) in order to provide space for Southeast Asian students navigating through their undergraduate studies. With this passion, they were accepted as an EMPOWER (Multicultural Retention Center) scholar where they learned the importance of researching within smaller communities. They applied to and were accepted into federally-funded training programs: National Institutes of HEALTH-funded BUILD EXITO at Portland State University and Center for Disease Control (CDC)-funded CUPS (CDC Undergraduate Public Health Scholars) at University of California - Los Angeles. These competitive research scholarships taught skills and reiterated the importance of culture within research. Prior research includes cognitive function, microbiome, and accessibility within rural areas. Angie currently works as a EXITO near-peer mentor and is a senior research assistant under Dr. Marriott, focusing on global inclusivity within LGBTQIA+ research.

Gwen Shusterman, PhD (she/her) is a Professor of Chemistry at Portland State University (PSU) teaching and working in the field of STEM education and equity. She was a first-generation college student entering a STEM discipline at a time when it was predominantly a male field, with many members carrying the bias that women did not belong. Coming from a family with a strong science and math-able female role model and with the help of mentors along the way, Gwen was able to navigate this environment and succeed. In her career at PSU, she has worked to level the playing field for students pursuing health professions through programs and advising designed to provide all students with the academic cultural capital to enhance their paths to professional programs. She co-created bridge programs for new and transfer first generation, low-income and multicultural students with Louis Stokes Alliance for Minority Participation in STEM (LSAMP), an NSF-funded S-STEM program (Reducing Transfer Shock: Developing Community and Collaborations to Support Urban STEM Transfer Students), and the PSU Transfer Center. With funding from a Howard Hughes Medical Institute Sustaining Excellence Grant and the American Public and Land-Grant University's Student Experience Project, Gwen has led cohorts of faculty and graduate students to develop and integrate pedagogical innovations in their classrooms to increase student sense of belonging and student success with a particular focus on those students traditionally marginalized in STEM fields. These funded projects also allowed the development at PSU, through a collaboration with theater faculty, the design and launch of the Equity Theater Project with a goal of growing awareness among privileged groups of the lived experience of others and to interrogate scenario options and actions by those observing bias and microaggressions. Gwen experienced the privilege of a family that was supportive of her path in higher ed and was able to financially assist her during undergrad years allowing her to imagine the further step to a graduate education and a PhD.
