# Supplementary material for: Inclusive Approaches for Measuring Demographics of Underrepresented Populations in STEM and Biomedical Research Training Programs
Source: J STEM Outreach. Author manuscript; Available in PMC 2024 Jul 12. (PMC11241867; doi:10.15695/jstem/v7i2.10)
Supplement: Parisetal2024_AppendixC_Operationalized [file NIHMS2004214-supplement-Parisetal2024_AppendixC_Operationalized.pdf]

Inclusive Demographics in Biomedical Training – Paris, et al.

Appendix C. Operationalized Phrasing for Demographic Categories

Table C1. *Overview of demographics recommended as a starting place for use in STEM training programs (Paris et al., 2021).*

Demographic Category	Benefits	Considerations
**Race and Ethnicity**	Race, Ethnicity, Language, and Disability (REALD) is a validated tool (McGee, 2020); Includes an open-ended prompt to start, then 34 response options. Permits data disaggregation and tracking of emergent populations. Mapped to NIH reporting categories (Paris et al., 2021).	More questions may be time-consuming to complete, particularly in program settings that are short in duration (e.g., museum interactions often encountered by informal science education programs). Small sample sizes can lead to privacy concerns; it may be easier to identify individuals within the dataset.
**Disability**	REALD (McGee, 2020) has 2-10 questions about hearing, vision, movement, communication, daily living, cognition, and mental health. Can be used for program accommodations and supports. Six of the seven disability questions are from the widely used American Community Survey (ACS; Brault, Stern, & Raglin, 2007).	The tool takes longer to complete than a single question but more descriptive on functional limitations. As described by McGee (2020): "These functional limitation questions were extensively tested to ensure that they work well to capture most people with disabilities. Notice how none of the questions relating to disability contain the word disability." Intersectional neurodiversity is not explicitly addressed.
**Disadvantaged Backgrounds**	Developed phrasing addresses NIH issued definitions (National Institutes of Health, 2019) to include 7 conditions related to socioeconomics and class (Paris et al., 2021; Huerta et al., 2022), including scoring criteria to merge rural variables	No clearly defined approach. Students underreport their eligibility when verified (Marriott et al., 2022). Some backgrounds are missing (e.g. immigrants, refugees); Rural geography is hard to capture for houseless individuals lacking addresses or for those with inconsistent zip codes due to frequent moving.
**Gender Identity and Gender Modality**	Open-ended prompts are most supportive of gender and sex diversities and can be qualitatively coded (see Paris et al., 2021; Marriott et al., 2021). If coding an open-ended prompt is not possible (i.e., large studies), we suggest using the two question approach from Morrison et al. (2021) that is inclusive of nonbinary, agender, and transgender individuals ("What is your current gender identity?" and "Are you transgender?").	Conflating sex and gender in research practices excludes entire populations whose physical characteristics and/or identities do not fit within the constraints of male/female. The erasure of sex and gender minority groups makes it impossible to provide services and accurate data for these populations. Treating sex and gender as independent variables allow researchers to collect more accurate data (Suen et al., 2020).
**Sexual Orientation Identity**	Single question: "Regardless of your sexual experience, how do you identify your sexual orientation?"; Includes options to select more than one item from the list; Options to self-describe support the fluidity and complexity of people's identities; provides a table containing sexual orientation definitions (Diamant et al., 2000)	Sexual orientation exists on a spectrum and is independent from gender identity. Research shows these populations face significant health disparities and discrimination. Programs should be mindful that the geopolitical setting of their STEM program will influence the strategic disclosure of student identities. Timing and development of sexual orientation varies among individuals; be mindful that responses may change.
**Language**	REALD (McGee, 2020) offers functional language questions for service-based systems and demographic language questions for non-service-based systems. All questions are suitable for individuals 5 years or older. The instrument is available in multiple languages (Oregon Health Authority, 2022).	Preferred language and English proficiency questions can be a predictor of an individual's ability to access services and programs. Populations with native languages other than English have historically been underrepresented in STEM and biomedical sciences. Communication barriers associated with limited English proficiency can lead to health inequities, including quality of care and adverse events.
**Religion**	Single question: "With which of the following do you currently identify?"; Includes ability to select more than one option to be inclusive of those with fluid belief systems or that incorporate multiple worldviews; Options to self-describe accommodate those whose beliefs may not align with the predefined categories.	The answer options provided might not be relevant or inclusive in all geographic regions, especially those outside of North America, therefore researchers and programs may need to adjust or add options reflective of the populations they are sampling (Hughes et al., 2022).
**Inclusive of Individuals Missed**	A great approach is the inclusion of the following question, with an open-field response option, "We realize we may have not captured everything about your background or experience. If you would like to say more, please feel free to share your story."	Research definitions of diversity evolve, reflecting the process of science. There are populations that remain excluded, marginalized, or improperly aggregated. We recommend being vigilant in looking for groups missing and including those individuals in program evaluation.

RACE AND ETHNICITY

Racial and ethnic minority groups often do not receive the same access to or quality of healthcare, resulting in avoidable health inequities (Baumann & Cabassa, 2020; Benson & Koroshetz, 2022). Greater resolution of data collection practices can help identify health inequities in sub-populations, guide development of culturally-specific and accessible services, and guide equitable allocation of resources to address inequities.

Recommended Instrument

We recommend the validated and tested Race, Ethnicity, Language, and Disability (REALD) instrument developed by Oregon Health Authority (OHA) (McGee, 2020), which is used throughout Oregon for demographic data collection. The following questions in Table C2 were taken verbatim from REALD (McGee, 2020; MSC 0074 rev.12/30/2020).

McGee, M.G. (2020). Race, ethnicity, language and disability (REALD) implementation guide. Portland, Oregon: Oregon Health Authority, Equity and Inclusion Division. (OHA Equity and Inclusion Division website; OHA REALD website; link to instrument (English - PDF; available in 43 other languages); and their excellent Implementation Guide PDF).

Benefits

- Race, Ethnicity, Language, and Disability (REALD) is a validated tool for collecting disaggregated demographic information on race and ethnicity, as well as language and disability (McGee, 2020).
- Includes an open-ended prompt to start, followed by 34 response options that permit data disaggregation and tracking of emergent populations.
- REALD has been mapped to NIH reporting categories (Paris et al., 2021) to support consistency and ease in federal reporting. https://www.startinstem.org/measures/demographics

Considerations

- More questions may be time-consuming to complete, particularly in program settings that are short in duration (e.g., museum interactions often encountered by informal science education programs).
- Small sample sizes can lead to privacy concerns; it may be easier to identify individuals within the dataset.

Table C2. *Operationalized phrasing for measuring racial and ethnic demographics (McGee, 2020; MSC 0074 rev.12/30/2020).*

Race & Ethnicity Question Wording	Rationale and Considerations
1. How do you identify your **race, ethnicity, tribal affiliation, country of origin, or ancestry**?<br>open field:__________________	"The intent of this question is to elicit an unprompted response. An open-ended question allows the client, respondent or member to identify the way they choose." (McGee et al., 2020)
2. Which of the following describes your **racial or ethnic identity**? Please check **ALL** that apply	"We also ask people to select their racial and ethnic identities from 34 options. Some options may be unfamiliar. There is an "other" category with space for the respondent to write in their response. There are a few situations that could require using the "other" category. Identities are fluid and personal. We want respondents to feel comfortable. This means providing an option to write in a racial or ethnic identity not listed. Doing so also helps us track emergent populations." (McGee et al., 2020)
**Hispanic and Latino/a/x**<br>Central American<br>Mexican<br>South American<br>Other Hispanic or Latino/a/x	Some equity researchers suggest expanding 'Latino/a/x' to 'Latino/a/x/e' to encompass the term 'Latine'. Language evolves over time as communities adopt new terms that better reflect their values and identities. It is important to respect the preferences and linguistic shifts that occur within a culture or community.
**Native Hawaiian and Pacific Islander**<br>CHamoru (Chamorro)<br>Marshallese<br>Communities of the Micronesian Region<br>Native Hawaiian<br>Samoan<br>Other Pacific Islander	Please note that the capitalized "CH" in CHamoru is intentional.
**White**<br>Eastern European<br>Slavic<br>Western European<br>Other White	
**American Indian and Alaska Native**<br>American Indian<br>Alaska Native<br>Canadian Inuit, Metis, or First Nation<br>Indigenous Mexican, Central American, or South American	

Table C2. *(Cont.)*

Race & Ethnicity Question Wording	Rationale and Considerations
**Black and African American**<br>African American<br>Afro-Caribbean<br>Ethiopian<br>Somali<br>Other African (Black)<br>Other Black	
**Middle Eastern/North African**<br>Middle Eastern<br>North African	
**Asian**<br>Asian Indian<br>Cambodian<br>Chinese<br>Communities of Myanmar<br>Filipino/a<br>Hmong<br>Japanese<br>Korean<br>Laotian<br>South Asian<br>Vietnamese<br>Other Asian	
**Other categories**<br>Other (please list) ___________<br>Don't know<br>Don't want to answer	
If given as a survey:<br>3. If you checked **more than one** category above, is there **one** you think of as your **primary** racial or ethnic identity?<br>Yes. Please circle your primary racial or ethnic identity above.<br>I do not have just one primary racial or ethnic identity.<br>No. I identify as Biracial or Multiracial. N/A. I only checked one category above. Don't know<br>Don't want to answer<br><br>Branching logic for electronic surveys:<br><If more than one option above is checked><br>3. You checked more than one category, is there **one** you think of as your **primary** racial or ethnic identity?<br>Yes. <present racial/ethic options again for single selection><br>I do not have just one primary racial or ethnic identity.<br>No. I identify as Biracial or Multiracial.<br>Don't knowDon't want to answer<br><br>Not shown due to branching logic<br>N/A. I only checked one category above.	"OARs 943-070-0000 through 943-070-0070 states that "Individuals who select multiple categories shall be asked an additional question regarding their primary racial or ethnic affiliation using the categories listed in section." By using response to the primary racial or ethnic identity question, you can avoid lumping everyone with more than one racial or ethnic identity as "multi" when you need to report in a way that results in unduplicated counts and percentages. The primary race and ethnic identity question helps us gain a more nuanced understanding of how persons identify based on lived experiences. That said, not everyone has just one primary racial or ethnic identity. Thus, it is important to allow people to indicate that they do not have just one primary identity if that is the case. For more about how people with two or more racial or ethnic identities may have more than one primary racial or ethnic identity, see 2015 Pew Research Center report (PDF) titled "Multiracial in America." In this report, 39 percent of persons who 24 Race, Ethnicity, Language, and Disability (REALD) Implementation Guide identified as having two or more races identified themselves as multi-racial (Pew Research Center, 2015)" (McGee et al., 2020)

Table C3. *Recommended mapping to federal funding agency demographic reporting forms (Paris et al., 2021).*

Primary Race/Ethnicity Reported by Participant	Coding to Federal Funding Agency Reporting Reporting
Hispanic or Latino/a/x	Hispanic or Latino
Native Hawaiian or Pacific Islander	Native Hawaiian or Pacific Islander
White	White
American Indian or Alaska Native	American Indian or Alaska Native
Black or African American	Black or African American
Middle Eastern/North African	Unknown or Not Reported[1]
Asian	Asian
Other categories	To be coded into demographic categories above as appropriate; use "Unknown or Not Reported" for "Don't Know" or "Prefer not to answer" responses.
I identify as Biracial or Multiracial.	More than one race[2]

[1]Middle Eastern and North African (MENA) individuals are categorized as White on U.S. Census forms (Office of Management and Budget, 2019; Federal Register #58782, 1997), yet represent diverse geographies and backgrounds who remain marginalized when categorized as White (Kayyali, 2013; Maghbouleh et al., 2022).

[2]Reporting racial/ethnic demographics on federal forms often does not permit nuanced identities, as the category "more than one race" can perpetuate underreporting due to data aggregation. For example, an underrepresented individual who identifies as both "Black" and "Native Hawaiian or Pacific Islander" would be categorized as "More than one race," masking their underrepresentation since they would be categorized in the same group ("More than one race") as an individual who identifies as "White" and "Asian," though the latter individual would not be considered underrepresented per NIH criteria (National Institutes of Health, 2019). When racial/ethnic demographic questions are not asked independently (i.e., select all that apply), they cannot be disaggregated, which can lead to underreporting of underrepresented populations. McGee (2020) recommends using the primary racial/ethnic identity (Table C2) to avoid unduplicated counts and percentages in federal reporting forms.

DISABILITY

Disabilities may be visible to others or unseen. Individuals with disabilities may experience discrimination and challenges living in a society not built for them, which can impact their health and quality of life (Hammell, 2015; Robertson, 2010), including how they engage in STEM education (Steele & Wolanin, 2004). Disability data collection can help mitigate preventable social and health inequities.

While disability measures can capture an individual's functional limitations, they may not fully capture the impact of social barriers on their lives, such as:

- A person with a physical disability may have difficulties entering a building due to the lack of power-assist doors on a building they need to access. Relating to STEM education, it may be more difficult for students with physical disabilities to travel long distances between training facilities.
- An individual with a mental health condition may face discrimination and stigma, which can limit their academic success or opportunities for career advancement. An individual may not be able to take a full time academic course load required by some federal training programs.
- Someone who has a chronic illness may experience financial hardship due to high medical expenses and a lack of access to affordable healthcare. An individual with such an experience may need to take an academic term off, which can be difficult for students who are financially dependent on stipends for their cost-of-living and livelihoods.

Social support is important for STEM students with disabilities.

- Disability Resource Centers offer services such as academic accommodations, assistive technology, and counseling services for students with disabilities.
- Peer mentors can help provide social and academic support, offering strategies for succeeding in STEM training programs across intersectional neurodiversities.
- Some schools may have social clubs or organizations for students with disabilities that would allow them to connect with others who share similar experiences and interests.

Recommended Instrument

We recommend the validated and tested Race, Ethnicity, Language, and Disability (REALD) instrument developed by Oregon Health Authority (McGee, 2020). The questions in Table C4 were taken verbatim from REALD (McGee, 2020; MSC 0074 rev.12/30/2020); Their excellent implementation guide is described and linked under "Race and Ethnicity."

Benefits

- REALD (McGee, 2020) has 2-10 questions about hearing, vision, movement, communication, daily living, cognition, and mental health.
- Six of the seven disability questions are from the widely used American Community Survey (ACS; Brault, Stern, & Raglin, 2007).
- Can be used for program accommodations and supports.

Considerations

- The tool is more descriptive but may be time-consuming to complete in some settings.
- Important note from McGee et al. (2020): The term "Health and Service Differences" is recommended for use in participant-facing materials instead of "disability". As described by McGee (2020): "These functional limitation questions were extensively tested to ensure that they work well to capture most people with disabilities. Notice how none of the questions relating to disability contain the word disability."
- The National Institutes of Health's Working Group on Diversity (WGD) Subgroup on Individuals with Disabilities published a report (2022) to define strategies in data collection and evidence-based practices for supporting individuals with disabilities (Bernard, 2022).

<u>Prompt for Participants</u>: "Your answers will help us find health and service differences among people with and without functional difficulties. Your answers are confidential."

Written surveys also include: (*Please write in "don't know" if you don't know when you acquired this condition, or "don't want to answer" if you don't want to answer the question.)"

Table C4. *Operationalized phrasing for measuring disability (McGee, 2020).*

Health & Service Differences Question Wording	Rationale
Are you **deaf** or do you have **serious difficulty hearing**?<br>Yes<br>[*If yes, at what age did this condition begin? ______]<br>No<br>Don't know<br>Don't want to answer	See Table 4 on page 38 of McGee (2020) for the underlying intent of question.
Are you **blind** or do you have **serious difficulty seeing**, even when wearing glasses?<br>Yes<br>[*If yes, at what age did this condition begin? ______]<br>No<br>Don't know<br>Don't want to answer	See Table 4 on page 38 of McGee (2020) for the underlying intent of question.
Please stop now if you/the person is under age 5	
Do you have **serious difficulty walking** or **climbing stairs**?<br>Yes<br>[*If yes, at what age did this condition begin? ______]<br>No<br>Don't know<br>Don't want to answer	See Table 4 on page 38 of McGee (2020) for the underlying intent of question.
Because of a physical, mental or emotional condition, do you have **serious difficulty concentrating**, **remembering,** or **making decisions**?<br>Yes<br>[*If yes, at what age did this condition begin? ______]<br>No<br>Don't know<br>Don't want to answer	See Table 4 on page 38 of McGee (2020) for the underlying intent of question.
Do you have **difficulty dressing** or **bathing**?<br>Yes<br>[*If yes, at what age did this condition begin? ______]<br>No<br>Don't know<br>Don't want to answer	See Table 4 on page 38 of McGee (2020) for the underlying intent of question.
Do you have **serious difficulty learning how to do things most people your age can learn**?<br>Yes<br>[*If yes, at what age did this condition begin? ______]<br>No<br>Don't know<br>Don't want to answer	See Table 4 on page 38 of McGee (2020) for the underlying intent of question.
Using your **usual** (**customary**) **language**, do you have **serious difficulty communicating** (for example understanding or being understood by others)?<br>Yes<br>[*If yes, at what age did this condition begin? ______]<br>No<br>Don't know<br>Don't want to answer<br>Don't know what this question is asking	See Table 4 on page 38 of McGee (2020) for the underlying intent of question.
Please stop now if you/the person is under age 15	
Because of a p**hysical, mental** or e**motional condition**, do you have **difficulty doing errands alone** such as visiting a doctor's office or shopping?<br>Yes<br>[*If yes, at what age did this condition begin? ______]<br>No<br>Don't know<br>Don't want to answer	See Table 4 on page 38 of McGee (2020) for the underlying intent of question.
Do you have **serious difficulty** with the following: **mood, intense feelings, controlling your behavior**, or **experiencing delusions or hallucinations**?<br>Yes<br>[*If yes, at what age did this condition begin? ______]<br>No<br>Don't know<br>Don't want to answer<br>Don't know what this question is asking	See Table 4 on page 38 of McGee (2020) for the underlying intent of question.

DISADVANTAGED BACKGROUND

Disadvantaged background often describes socioeconomic or environmental conditions that impact access to education or training environments. Disadvantaged background definitions have evolved over time. NIH issued definitions in 2018 and changed them in 2019 (National Institutes of Health, 2018, 2019). NIH variables for disadvantaged background largely relate to socioeconomics and class. Of the following items, those denoted by an asterisk were underreported by students when verified (Marriott et al., 2022).

- Experienced past or present homelessness
- Previously or presently in the foster care system
- Eligible for the Federal Free and Reduced Lunch Program for 2+ years
- First generation college students*
- Eligible for Federal Pell grants
- Received WIC as a parent or child
- Grew up in a rural area defined by either Health Resources and Services Administration (HRSA) address* or Health Professional Shortage Area (HPSA)* locations (zip code used for eligibility)

Recommended Instrument

We have not found a validated instrument for asking questions about disadvantaged background. The following questions in Table C5 were developed based on National Institutes of Health (NIH) categories used to define disadvantaged background (see NOT-OD-020-031; National Institutes of Health, 2019).

Benefits

- Phrasing addresses NIH-issued definitions of disadvantaged background (National Institutes of Health, 2019) and includes seven conditions related to socioeconomics and class (Paris et al., 2021), including scoring criteria that enables merging of rural variables and generation of a composite score.

Considerations

- There is no clearly defined approach.
- Students underreport their eligibility when verified (Marriott et al., 2022; Huerta et al., 2022).
- Some disadvantaged backgrounds remain missing (e.g. immigrants, refugees).
- Rural geography is hard to capture for houseless individuals lacking addresses or for those with inconsistent zip codes due to frequent moving (e.g., houseless individuals, families with military backgrounds).
- We highly recommend supplementing these questions with phrasing from Marr (2021): "We realize we may have not captured everything about your background or experience. If you would like to say more, please feel free to share your story" [open-field] (Marr, 2021).

Participant Prompt: Some groups have been historically underrepresented in science, technology, engineering, and math fields. Please check all areas of diversity that apply or have applied to you (some items may have been true in the past). If you are not sure, you can indicate "I am not sure" or leave blank.

Table C5. *Operationalized phrasing for measuring disadvantaged backgrounds identified by (National Institutes of Health, 2019). Phrasing developed by Marr and Marriott (Paris et al., 2021).*

Disadvantaged Background Question Wording	Rationale
Are you a first generation college student? (i.e., the first person in your family to attend college and receive a bachelor's degree. Older siblings going to college does not disqualify you as a first generation college student.)<br>Yes<br>No<br>Don't know<br>Prefer not to say	Variable used to define NIH underrepresented student eligibility. Describes students who have/had no parents or legal guardians who completed a bachelor's degree (Redford & Mulvaney Hoyer, 2017; see https://nces.ed.gov/pubs2018/2018009.pdf)
Parent/Guardian 1 educational level<br>• Less than high school degree<br>• High School diploma or GED (General Education Development) equivalent<br>• Some college (no degree)<br>• Associate's degree (AA, AAS, AAOT, etc.)<br>• Bachelor's Degree (BA, BS)<br>• Some graduate training<br>• Master's Degree (MA, MS, MAT, etc.)<br>• Doctoral Degree (MD, PhD, DDT, DMD, PharmD)<br><br>Parent/Guardian 2 educational level<br>• Less than high school degree<br>• High School diploma or GED (General Education Development) equivalent<br>• Some college (no degree)<br>• Associate's degree (AA, AAS, AAOT, etc.)<br>• Bachelor's Degree (BA, BS)<br>• Some graduate training<br>• Master's Degree (MA, MS, MAT, etc.)<br>• Doctoral Degree (MD, PhD, DDT, DMD, PharmD)	Recommended adding to support verification by program staff due to underreporting of first generation college student status by students (Marriott et al., 2022; Huerta et al., 2022)
Did you grow up in a U.S. rural area?<br>Yes<br>No<br>Don't know<br>Prefer not to say<br><br>U.S. rural area eligibility:<br>https://data.hrsa.gov/tools/rural-health<br><br>Address:________________________________ [OR]<br>Zip code:________________________________	Variable used to define NIH underrepresented student eligibility. A U.S. rural area is defined by the Health Resources and Services Administration (n.d.; HRSA) Rural Health Grants Eligibility Analyzer (https://data.hrsa.gov/tools/rural-health) based on address. Zip code can be used to approximate, since address and zip code are HIPAA identifiers which influence their use across settings (U.S. Department of Health & Human Services, n.d.). Please note that students have difficulty assessing their rural geography and underreport rural eligibility when verified with address (Marriott et al., 2022). Address enables exact verification while zip code provides an approximation. Often, data from student applications to programs can be used to verify this variable. Rural geography determined by rural (HRSA) OR HPSA eligibility (next question).
Did you grow up in a medically underserved area?<br>Yes<br>No<br>Don't know<br>Prefer not to say<br><br>Eligibility: Centers for Medicare and Medicaid Services-designated Low-Income and Health Professional Shortage Areas<br><br>Zip code:______________________________	Used to define rural eligibility through HRSA (above) OR HPSA (this question) eligibility. Rural is a single variable used to define NIH underrepresented eligibility. Assessed using qualifying zip codes provided in an excel file (Centers for Medicare and Medicaid Services, 2020). Please note that students have difficulty assessing their rural geography and underreport rural eligibility when verified with address (Marriott et al., 2022). Address enables exact verification while zip code provides an approximation. Often, data from student applications to programs can be used to verify this variable.
Did you grow up with a low socioeconomic background?<br>Yes<br>No<br>Don't know<br>Prefer not to say	This question is used as an indicator variable for disadvantaged background for students who may not know answers to the questions below. It is not one of the composite variables used to officially categorize disadvantaged background. Younger students may not know how to answer this question.
Have you ever been eligible for Federal Free and Reduced Lunch Program for two or more years?<br>Yes<br>No<br>Don't know<br>Prefer not to say	Variable used to define NIH underrepresented student eligibility. Student describes eligibility for Federal Free and Reduced Lunch Program for two or more years (U.S. Department of Agriculture Food and Nutrition Service (2023a; see https://www.fns.usda.gov/school-meals/income-eligibility-guidelines). Younger students may not know how to answer this question.
Have you ever received support from Special Supplemental Nutrition Program for Women, Infants, and Children (WIC) as a parent or child? (Eligibility: https://www.fns.usda.gov/wic/wic-eligibility-requirements)<br>Yes<br>No<br>Don't know<br>Prefer not to say	Variable used to define NIH underrepresented student eligibility. Includes students who currently or previously received support from Special Supplemental Nutrition Program for Women, Infants, and Children (WIC) as a parent or a child (U.S. Department of Agriculture Food and Nutrition Service (2023b; see https://www.fns.usda.gov/wic/wic-eligibility-requirements). Younger students may not know how to answer this question.

Table C5. *(Cont.)*

Disadvantaged Background Question Wording	Rationale
Have you ever been eligible for federal Pell grants?<br>(Definition: https://www2.ed.gov/programs/fpg/eligibility.html)<br>Yes<br>No<br>Don't know<br>Prefer not to say	Variable used to define NIH underrepresented student eligibility. Student was or is currently eligible for federal Pell grants (U.S. Department of Education, 2022; see: https://www2.ed.gov/programs/fpg/eligibility.html). Younger students may not know how to answer this question.
Have you ever been in the foster care system as an infant/child/adolescent, as defined by the Administration for Children and Families?<br>(Definition: https://www.acf.hhs.gov/cb/focus-areas/foster-care)<br>Yes<br>No<br>Don't know<br>Prefer not to say	Variable used to define NIH underrepresented student eligibility. Student was or currently is in the foster care system as an infant, child, or adolescent, as defined by the Administration for Children and Families (National Institutes of Health, (2019) references https://www.acf.hhs.gov/cb/focus-areas/foster-care), also described at Child Welfare Information Gateway (n.d.)
Have you ever experienced houselessness/housing instability, as defined by the McKinney-Vento Homeless Assistance Act?<br>(Definition: https://nche.ed.gov/mckinney-vento/)<br>Yes<br>No<br>Don't know<br>Prefer not to say	Variable used to define NIH underrepresented student eligibility. Describes student who experienced houselessness/housing instability at any time, as defined by the McKinney-Vento Homeless Assistance Act (National Center for Homeless Education (n.d.); see https://nche.ed.gov/mckinney-vento.

GENDER IDENTITY AND GENDER MODALITY

Although gender has historically been defined as masculine or feminine, there is significant variation, fluidity, and nuances of gender identity and gender expression. Conflating sex and gender in research practices can exclude entire populations whose physical characteristics and/or identities do not fit within the constraints of male/female (Ashley, 2022; Heidari et al., 2016). The erasure of sex and gender minority groups makes it impossible to provide services and accurate data for these populations (Morrison et al., 2021). Treating sex and gender as independent variables allows researchers to collect more accurate data (Suen et al., 2020). SSGM is an acronym that describes sex minorities, sexual orientation minorities, and gender minorities. Things to remember:

- **Terminology gaps interfere with people's ability to speak about important realities and lived experiences.** There is no categorical term that describes an individual's experience being cis or trans, which inherently enforces the cis/trans binary (Ashley, 2020; Hart et al., 2019). Ashley (2020) proposes the adoption of a new term, gender modality, an open-ended category that refers to how a person's gender identity stands in relation to their gender assigned at birth, and includes being cis, trans, and any other emergent term that describes the experiences people may have of the relationship between their gender identity and their gender assigned at birth.
- **Inclusive measures for gender identity allow for accurate data collection of populations who may use culturally-specific linguistic terms to define gender.** Some populations use a culturally distinct "third" or nonbinary gender identities, such as fa'afafine in Samoan, Māhūwahine in Native Hawaiian, or Nádleehi in Navajo/Diné (National Academies of Sciences, Engineering, and Medicine, 2022).

Recommended Instrument

We recommend two approaches, which can be used depending on the size of the STEM program or research study. While use of an open-ended prompt is most inclusive (see option 1 below), we recognize that it can be challenging for larger studies since the data will take more time to qualitatively code. For larger programs and studies, we recommend the two question approach from Morrison et al. (2021) that is inclusive of nonbinary, agender, and transgender individuals (see option 2 below; defined in Table C6). Table C6 applies gender phrasing defined by Oregon Health Authority Office of Equity and Inclusion (2022).

Benefits

- Open-ended prompts are most supportive of gender and sex diversities and can be qualitatively coded (Marriott et al., 2021; Paris et al., 2021).
- For large studies, we suggest using the two question approach from Morrison and colleagues (2021) that is inclusive of nonbinary, agender, and transgender individuals. It matches the approach currently in development for use statewide (Oregon Health Authority Office of Equity and Inclusion, 2022).

Considerations

- Studies should include an option to self-describe even if the two question approach is selected.
- Instead of using the term "other," replace it with more inclusive terms such as "prefer to self-describe" with an open-field option.
- Be mindful of your audience, as inclusion of culturally-appropriate linguistic terms can be used to enhance students feeling welcome in your STEM education program. Remember that data collection is about welcoming individuals, which is different from reporting summarized data when grouping may take place to support privacy of SSGM individuals.
- Pronouns are another way to make students feel more welcomed, though they should be optional, just as any other demographic field would be.
- Consider whether sex-assigned-at-birth is actually needed for your project, as gender identity may be more inclusive for students in your programs.
- The National Institutes of Health Sexual & Gender Minority Research Office (2020) has summarized other approaches used across research studies; see https://dpcpsi.nih.gov/sgmro/measurement/questions

Option 1: Open-Ended

When possible, use open-ended prompts that you can subsequently qualitatively code into categories described in Table C6 for reporting. Open-ended prompts are most supportive of diverse gender identities.

What is your gender? ________________________

Option 2: Prompted Questions

In larger studies where coding an open-ended prompt is challenging, we suggest using the two-question approach from Morrison et al. (2021) and OHSU Evaluation Core (2019), using phrasing from the Oregon Health Authority's Office of Equity and Inclusion statewide SOGI measure currently in development (2022).

Table C6. *Operationalized phrasing for measuring gender identity.*

Question Wording	Rationale
What is your gender identity (select all that apply):<br>• Girl/Woman/Feminine<br>• Boy/Man/Masculine<br>• Non-binary<br>• Agender/No gender<br>• Questioning<br>• Prefer to self-describe:________<br>• Prefer not to answer<br>• I don't know what this question is asking<br><br>Are you transgender?<br>• Yes<br>• No<br>• Questioning<br>• Prefer not to answer<br>• I don't know what this question is asking	These questions are intended to support accurate identification and inclusive data collection for sex and gender minorities (SGMs). Options exist to select more than one option and/or self-describe to support fluidity and complexity of people's identities. Additional inclusive options may be added (e.g., Genderfluid, Gender Queer). Please notice how "other" is not included within answer options.<br><br>"Another identity:________" can be used as an alternative to the original prompt "Prefer to self-describe:________" for the write-in option. This choice of wording acknowledges that the list of identities provided is incomplete, and may encourage participants to express their unique identity.<br><br>Younger students may have difficulty answering questions, which is why Oregon Health Authority's Office of Equity and Inclusion (2022) recommends including an option "I don't know what this question is asking." which captures comprehension difficulties with the question and/or response options rather than "Don't know", which they include and would be used if questions were being completed by a parent answering for a child.<br><br>The term "Two-Spirit" is a culturally-specific pan-North American Indigenous "third gender" term. It should be offered as a structured gender identity choice on the survey only when individuals have indicated that they identify as some North American Indigenous category under race and ethnicity. This practice aims to avoid inviting appropriation by individuals outside this broad community.<br><br>A glossary of definitions can be provided (see examples in Table C7 or Morrison et al., 2021).

Table C7. *Glossary of gender definitions (Suen et al., 2020; Ashley, 2022).*

Term	Definition
Transgender woman	A person who identifies as a woman and was assigned masculine gender at birth
Transgender man	A person who identifies as a man and was assigned feminine gender at birth
Transgender/trans	A person with a gender identity that differs from that commonly associated with their gender assigned at birth
Sex assigned at birth	The sex assigned to each person at time of birth or shortly thereafter usually based on external genitalia, also referred to as natal sex or biologic sex. This describes anatomic and/or physiologic characteristics
Intersex	A person who is born with any of a range of sex characteristics that may not fit typical notions of binary "male" or "female" bodies. Sometimes used to describe people who have differences of sex development
Genderqueer	A gender identity usually used in one of two ways: (1) as an umbrella term that includes all people whose gender identity varies from the traditional cultural notions of gender; or (2) to describe a person whose gender identity does not fully fall along the gender binary of being girl/woman or boy/man, similar to gender non-binary
Gender non-conforming	A person whose gender identity or expression does not fully conform to sex-linked social expectations (e.g., masculine girls/women, feminine boys/men)
Gender non-binary	A person whose gender identity does not fully fall along the gender binary of being a girl/woman or boy/man
Gender minority	A person with a gender identity that differs from that commonly associated with their sex assigned at birth
Gender identity	A person's deeply-felt, self-conceptualization of being a boy, a man, or male; a girl, a woman, or female; or another gender (e.g., genderqueer, gender nonconforming, agender/no gender) that may or may not correspond to that commonly associated with a person's sex assigned at birth or to a person's primary or secondary sex characteristics
Gender expression	Characteristics in a person's appearance, personality, and behavior that are culturally and temporally defined as masculine, feminine, or outside of the masculine or feminine binary
Cisgender	A person with a current gender identity that conforms to the gender they were assigned at birth

Adapted from Table 1 in Suen et al. (2020; https://link.springer.com/article/10.1007/s10508-020-01810-y/tables/1); with updates and additions from Ashley (2022; https://www.florenceashley.com/uploads/1/2/4/4/124439164/ashley_trans_is_my_gender_modality.pdf).

Coding gender to federal funding agency inclusion and reporting tables

With the recommendation to include open-ended prompts, it feels prudent to offer guidance on how to code the resulting data. In prior work, colleagues at the OHSU Evaluation Core worked with a partner organization whose mission was to make schools and communities safer for people of all sexual orientations and gender identities. A total of 33 gender identity response options were collected from 168 individuals (OHSU Evaluation Core, 2019), which were transparently condensed into categories described in Table C6. They offer recommendations for replicating their process. Currently the National Institutes of Health asks for inclusion and enrollment tables for participants in federally-funded STEM training programs, though their reporting options include only female, male, and unknown/not reported. Therefore, SSGM individuals are underreported in federal funding agency reporting. We recommend the mapping described in Table C8 to support STEM training programs reporting to federal agencies.

Table C8. *Recommended mapping to NIH demographics (Paris et al., 2021).*

Gender Identity Reported by Participant	Coding to NIH Reporting Forms	Notes and Rationale
Girl/Woman/Feminine	Female	Honors the gender identity of the student
Boy/Man/Masculine	Male	Honors the gender identity of the student
Non-binary	Unknown/Not reported	Currently, NIH forms do not have options beyond the binary. Therefore, "unknown/not reported" is the closest current option. Will adjust as NIH reporting categories expand.
Agender/No gender	Unknown/Not reported	See notes and rationale for non-binary
Prefer to self-describe (open-field)	Unknown/Not reported	Initial coding as unknown/not reported until qualitative coding can occur; see OHSU Evaluation Core (2019); https://www.ohsu.edu/sites/default/files/2020-01/OCTRI_PDF_Evaluation_Measuring%20SOGI_2020%2001%2016.pdf
Questioning	Unknown/Not reported	Indicates that an individual is questioning their gender identity. See notes and rationale for non-binary.
Prefer not to answer	Unknown/Not reported	N/A
I don't know what this question is asking	Unknown/Not reported	Supports understanding of ages where gender identity questions are understandable to youth in STEM training programs
Transgender	Use gender defined by the student/participant<br>Girl/woman/feminine = Female<br>Boy/man/masculine = Male<br>Non-binary, agender/no gender, and questioning = Unknown/ Not reported	Transgender denotes gender assigned at birth is not the same as current gender identity (Ashley, 2022). We recommend honoring the self-identified gender of individuals in STEM education programs, regardless of whether the student is cis- or transgender. Data collection on transgender students in STEM education programs supports documentation of prevalence needed to mitigate erasure of transgender student experiences, identify inequities in training, and more accurately represent both cisgender and transgender experiences in gender identity categories.
Cisgender	See gender identity options above	Cisgender denotes 'Not transgender', i.e. gender assigned at birth conforms to current gender identity.

SEXUAL ORIENTATION IDENTITY

Sexual orientation exists on a spectrum and is independent from gender identity. The two are often conflated in research studies. Measuring sexual orientation separately from gender and sex provides more accurate representation of populations. Sexual orientation is not included among NIH definitions of underrepresentation despite significant bias, discrimination, and inequities faced by LGBTQIA+ students (Campbell-Montalvo et al., 2022a; Cech & Waidzunas, 2021; Freeman, 2018; Freeman, 2020; Hughes, 2018; Marr et al.,2022; Palmer et al., 2022). In our prior work, only 7% of training programs we sampled asked students about sexual orientation (Mekinda et al., 2022). Further inclusion is warranted (Mapes et al., 2020; White House, 2021a, 2021b, 2022, 2023).

We recognize that questions asking about sexual orientation can be viewed as private and age-sensitive. We fully recognize that some of the sexual orientation continuums may be viewed as outside the evaluation scope of a STEM education program, particularly for younger students. However, as inclusive demographics aim to make individuals feel welcome, we encourage STEM training programs to be familiar with terms and how they could make room for open-ended prompts to be inclusive of students with diverse sexual orientation identities in their programs. As general guidance, programs should be familiar with the following:

<u>Audience Age and Safety</u>

- Sexual orientation identity can be asked in ways that do not imply sexual experience.
- Students of younger ages may not yet be aware of sexual attraction or have a sense of their sexual orientation identity. Colleagues are studying language, such as the term "crush" for use with younger audiences. Likewise, the Oregon Health Authority is studying appropriate ages for asking about sexual orientation identity. Our current recommendation is to include it with middle school audiences and older.
- That said, programs should balance age with question content, being mindful to avoid harming individuals in the process. In the context of parents or other caregivers for minors, sexual orientation minority or gender minority minors may or may not be out to their caregivers, who may be supportive, non-supportive, or actively hostile.

- There are likewise intersections with legal systems in varying contexts where minors are or are not guaranteed confidentiality of their demographics from their parents/caregivers, and varying contexts where parents/caregivers are or are not guaranteed access to their minors' demographic information.
- We urge STEM programs to protect their students' safety, privacy, and autonomy.

<u>Sexual Orientation Variable Measurement and Continuums</u>

- Reflect thoughtfully on representation and variables that address underrepresentation, recognizing that some demographic categories may be in greater depth or outside project scope, depending on audience age.
- Use caution to not conflate sexual orientation with other variables, such as desire, behavior, relationship structure, self-identity, or community affiliation. For example, there are multiple continuums on which sexual orientation can be measured beyond LGBTQIA+, such as asexual to sexual; aromantic to romantic; monosexual-polysexual; identification with categories of kink and of power exchange; acceptable differences in age relative to one's prospective or actual partners, etc.
- Likewise, marital status can impact tax implications for trainees in STEM education programs (particularly at undergraduate and graduate levels), though marital status is rarely included in STEM training program demographics. While marital demographics may be viewed as straightforward (e.g., married, single, divorced, widowed, etc.), we want to remind STEM programs that individuals being able to choose a category that fits their own experience is more supportive of their trainees, as individuals within "marital status" may be non-monogamous or polyamorous, which have their own sub-sects and continuums.
- These spectra reinforce why open-ended prompts can be helpful and inclusive of SSGM students.

Recommended Instrument

Given the focus of STEM education programs working with younger audiences, we selected question and answer responses adapted from Diamant et al. (2000) and Katz-Wise et al. (2016) based on advice provided in Suen et al. (2020) to measure sexual orientation identity. Specifically, we liked the introduction language "regardless of your sexual experience", which felt more neutral for use with students in our programs. We are currently using phrasing described in Table C9, but are closely monitoring the instrument in development by the Oregon Health Authority's Office of Equity and Inclusion (2022) described in Table C10. Their large-scale testing of the instrument across ages and statewide settings will inform our future practices, underscoring the iterative nature of demographic instruments and adaptations to be more inclusive and equitable.

Benefits

- Single question: "Regardless of your sexual experience, how do you identify your sexual orientation?"
- Includes ability to select more than one option from the list to support measurement across multiple continuums.
- Options to self-describe support the fluidity and complexity of people's identities, which we recognize may change over time.
- A table describing sexual orientation definitions can be provided (Suen et al., 2000; Table C11).

Considerations

- Timing and development of sexual orientation varies among individuals; it is recommended that programs ask about sexual orientation over the course of their programs, being mindful that responses may change.
- Programs should be mindful that the geopolitical setting of their STEM program will influence the strategic disclosure of student identities, as some students may not feel safe disclosing their identities.

Table C9. *Operationalized phrasing for measuring sexual orientation identity (Paris et al., 2021).*

Sexual Orientation Identity Question Wording	Rationale
Regardless of your sexual experience, how do you identify your sexual orientation? (select ALL that apply)<br><br>Straight or heterosexual<br>Gay<br>Lesbian<br>Bisexual<br>Pansexual<br>Asexual<br>Queer<br>Fluid<br>Questioning<br>Prefer to self-describe: ______<br>Prefer not to say<br>I don't know what this question is asking	This question uses question and answer responses adapted from Diamant et al. (2000) and Katz-Wise et al. (2016) based on advice provided in Suen et al. (2020) to measure sexual orientation identity. It is intended to support accurate identification and inclusive data collection for SSGM individuals.<br><br>Options exist to select more than one option and/or self-describe to support fluidity and complexity of people's identities. A glossary of definitions can be provided (see Table C11).<br><br>An alternate approach currently in development for use with a broad age range in medical/public health settings is described in Table C10.

Alternatively, Table C10 illustrates the question and answer responses currently in development by the Oregon Health Authority's Office of Equity and Inclusion (2022) to support the accurate measure of sexual orientation across a wider spectrum of settings. Their instruments and data collection recommendations were updated in November 2023).

- Draft Instrument (September 2022, updated in November 2023): https://www.oregon.gov/oha/EI/REALD%20Documents/DRAFT-SOGI-Recommendations.pdf
- Website for following their work and signing up for update notifications as items are developed: https://www.oregon.gov/oha/EI/Pages/Demographics.aspx
- An implementation guide is not yet developed as their instrument is still in progress

In the draft instrument (2022), they also include logistical questions applicable for social services and eligibility systems (see their Figure 2) as well as best practice recommendations to assure quality medical care (e.g., questions related to sexual health, body anatomy, and trans health). While not all of these questions may be needed for measuring the demographics of students in STEM education programs, having familiarity with these questions and the settings in which they can be used may be helpful to partners who work across service settings (e.g., STEM program staff who may also be medical providers, public health practitioners, researchers conducting relevant studies, etc.).

Table C10. *Operationalized phrasing for measuring sexual orientation identity in their draft instrument (Oregon Health Authority Office of Equity and Inclusion, 2022).*

Question Wording	Rationale
How do you describe your sexual orientation or sexual identity? (check all that apply)<br><br>Same-gender loving<br>Same-sex loving<br>Lesbian<br>Gay<br>Bisexual<br>Pansexual<br>Straight (attracted mainly to or only to other gender(s) or sex(s))<br>Asexual<br>Queer<br>Questioning<br>Don't know<br>Not listed.<br>Please specify: ______________________<br>I don't know what this question is asking<br>I don't want to answer	This question is currently in development by the Oregon Health Authority Office of Equity and Inclusion's Sexual Orientation and Gender Identity (SOGI) Data Collection Workgroup to measure sexual orientation or sexual identity. The group consists of individuals within and outside the OHA "who interact with the LGBTQ+ community and health systems in a myriad of ways, many of whom also identify as LGBTQ+ themselves" (Oregon Health Authority Office of Equity and Inclusion, 2022).<br><br>Additional questions for social services and medical care also provided (2022): https://www.oregon.gov/oha/EI/REALD%20Documents/DRAFT-SOGI-Recommendations.pdf

Table C11. *Glossary of sexual orientation definitions (Suen et al., 2020).*

Term	Definition
Asexual	A sexual identity describing people who do not experience sexual attraction to people of any gender but may still have romantic attractions to other people
Bisexual	A sexual identity where sexual attractions and/or behaviors are focused on members of both sexes (usually female and male) or gender identities (women and men). Increasingly this is used to describe people whose sexual attractions and/or behaviors are with people of the same and/or another gender
Gay	A sexual identity where sexual attractions and/or behaviors are focused mainly on members of the same gender identity
Graysexual/demisexual	A sexual identity where sexual attractions occur only occasionally and under specific circumstances, usually after developing a very strong bond
Heterosexual/straight	A sexual identity where attractions and/or behaviors are focused mainly on members of another gender identity
Lesbian	A sexual identity where attractions and/or behaviors are focused mainly on members of the same gender identity, usually referencing those who identify as women
Pansexual	A sexual identity where sexual attractions can occur toward individuals of all gender identities or expressions
Sexual minority	A person with a sexual identity that is not strictly straight or heterosexual
Sexual orientation	An enduring pattern of emotional, romantic, and/or sexual attractions; a person's sense of identity based on those attractions, related behaviors, and membership in a community of others who share those attractions

Excerpted from Table 1 in Suen et al. (2020); https://link.springer.com/article/10.1007/s10508-020-01810-y/tables/1.

Table C12. *Mapping of sexual orientation identity to reporting variables.*

Sexual Orientation Identity Question Wording	Coding	Rationale
Straight or heterosexual	Straight	
Any option below:<br>Gay<br>Lesbian<br>Bisexual<br>Pansexual<br>Asexual<br>Queer<br>Fluid	LGBTQIA	LGBTQIA+ [note "+" is removed from variable coding due to incompatibility in software programs]<br><br>*Transgender is included within LGBTQIA+ and sourced from in the gender identity question table
Questioning	Unknown or not reported	Individual has not indicated a sexual orientation identity. Highlights why SOGI questions can/should be asked over time. The author team also recognizes that this answer option can be considered within the LGBTQIA+ spectrum, as fluidity exists when considering identity. An inclusive approach would be to consider this category within the LGBTQIA+ spectrum, particularly when answered in combination with another LGBTQIA+ response.
Prefer to self-describe:____	Unknown or not reported	Response may reflect LGBTQIA+, but may be excluded from initial data reporting if not coded in time. Will be immediately coded as "unknown or not reported", as the response needs to be qualitatively coded for accuracy; see OHSU Evaluation Core (2019); https://www.ohsu.edu/sites/default/files/2020-01/OCTRI_PDF_Evaluation_Measuring%20SOGI_2020%2001%2016.pdf
Prefer not to say	Unknown or not reported	Denotes preference for not disclosing information
I don't know what this question is asking	Unknown or not reported	Denotes confusion about question; not orientation questioning
More than one response (includes any response that would be LGBTQIA+ coded)	LGBTQIA	Individuals who select more than one option within the LGBTQIA+ spectrum, described above, to support representation (e.g., "lesbian" AND "queer").<br><br>An inclusive approach would be to also include multiple responses that include at least one selection that is LGBTQIA+ for representation purposes (e.g., "Fluid" AND "Prefer not to say", "Asexual" AND "Questioning", "Gay" AND "Straight"), since there is at least one of the LGBTQIA+ answer options chosen. There was team discussion about what combinations should be flagged for further review and coded as "unknown or not reported" (e.g., selecting every response option).
More than one response (includes responses that would be coded as "unknown or not reported")	Unknown or not reported	If an individual selects multiple options where the combination presents uncertainty for reporting, responses can conservatively be reported as "unknown or not reported" (e.g., "Straight" AND "Prefer not to say", "Straight" AND "I don't know what this question is asking", etc.). It is important to note automatically coding "questioning", "I don't know what this question is asking", and/or "Prefer not to say" as "Unknown or not reported" will likely underrepresent the LGBTQIA+ community within the data.

LANGUAGE

Communication barriers associated with limited English proficiency can lead to health inequities, including quality of care and adverse medical events that result in physical harm (Ngo-Metzger et al., 2003; Timmins, 2002; Ulmer et al., 2009). Populations with native languages other than English may face more challenges in STEM and biomedical sciences as they translate complex STEM terms.

Recommended Instrument

We recommend the validated and tested Race, Ethnicity, Language, and Disability (REALD) instrument developed by Oregon Health Authority (McGee, 2020), which is used throughout Oregon for demographic data collection. The following questions in Table C13 were taken verbatim from REALD (McGee, 2020; MSC 0074 rev.12/30/2020; see Table 3b on page 35). We recommend the version for non-service based settings for use with STEM education programs (Table C13)

Benefits

- REALD (McGee, 2020) offers functional language questions for service-based systems and demographic language questions for non-service-based systems.
- All questions are suitable for individuals 5 years or older.
- The instrument is available in multiple languages (Oregon Health Authority, 2022; website: https://www.oregon.gov/oha/EI/Pages/Demographics.aspx)

Considerations

- Preferred language and English proficiency questions can be a predictor of an individual's ability to access services and programs, including those in STEM.
- The survey can be used to understand what languages program materials should be translated into for supporting communication with students and their families.

Table C13. *Language questions within the REALD (McGee, 2020).*

Language Question Wording	Rationale
1a. Do you speak a language other than English at home?<br>Yes<br>No	
1b. If yes, what is this language?<br>____________________	
If yes to #1a above:	
2a. In what language do you prefer to use when speaking with someone outside the home about important matters (such as medical/legal/health information)<br>____________________	
2b. In what language do you prefer to use when reading important matters (such as medical/legal/health information)?<br>____________________	
3. How well do you speak English?<br>Very well<br>Well<br>Not well<br>Not at all<br>Don't know<br>Don't want to answer	

RELIGION

Recommended Instrument

We recommend the question and answer responses in Table C14, adapted from American College Personnel Association (2022) and Hughes et al. (2022). This recommendation uses a broad question that allows those with theistic faiths, spiritual nontheistic beliefs, and nonspiritual worldviews to be captured within one demographic question.

Benefits

- Single question: "With which of the following do you currently identify?"
- Includes ability to select more than one option from the list to be inclusive of those with fluid belief systems or that incorporate multiple worldviews.
- Options to self-describe accommodate those whose beliefs may not align with the predefined categories.

Considerations

The answer options provided might not be relevant or inclusive in all geographic regions, especially those outside of North America, therefore researchers and programs may need to adjust or add options reflective of the populations they are sampling (Hughes et al., 2022).

Table C14. *Operationalized phrasing for measuring religion.*

Question Wording	Rationale
With which of the following do you currently identify?<br>(Please check ALL that apply)<br>• Agnostic<br>• Animist<br>• Atheist<br>• Baha'i<br>• Buddhist<br>• Christian (Including other descriptions which might include related faith or practice communities)<br>• Deist<br>• Hindu<br>• Humanist<br>• Jewish (Including other descriptions which might include related faith or practice communities)<br>• Muslim<br>• Pagan<br>• Pantheist<br>• Polytheist<br>• Secular<br>• Sikh<br>• Spiritual but not religious<br>• Taoist<br>• Unitarian Universalist<br>• Wiccan<br>• Don't know<br>• Prefer to self-describe:________<br>• I don't know what this question is asking<br>• I don't want to answer	This question has been adapted from question D of the ACPA Standards for Demographic Questions (American College Personnel Association, 2022) as well as wording suggestions from Hughes et al. (2022).

CAPTURING ADDITIONAL FIELDS

Research definitions of diversity evolve, reflecting the process of science. There are still populations that are being excluded, marginalized, or improperly aggregated. We highly recommend that programs look to include these student populations. A great approach is the inclusion of the following question, with an open-field response option (Marr, 2021):

> **“We realize we may have not captured everything about your background or experience. If you would like to say more, please feel free to share your story”**

Considerations

Mapes et al. (2020) describe barriers to health that are intersectional to STEM education and training, such as:

- Financial (e.g., insurance coverage, income)
- Cognitive (e.g., health literacy, technical proficiency)
- Medical access (e.g., Individuals who have not had a needed medical visit in the past 12 months or cannot easily obtain or pay for medical care as needed)
- Communication and cultural factors (e.g., cultural humility, lack of trust)
- Structural (e.g., transportation, geographic accessibility)
- Racial and ethnic discrimination (see section above)
- Language (e.g., lack of translation resources; see section above)
- Study design (e.g., eligibility criteria limiting participation or inclusion, failure to reach diverse groups due to recruitment limitations)

We urge STEM programs to be diligent in looking for populations missing in their STEM evaluation and to continue to find ways to improve the STEM training environments for their students.
