# Supplementary material for: Inclusive Approaches for Measuring Demographics of Underrepresented Populations in STEM and Biomedical Research Training Programs
Source: J STEM Outreach. Author manuscript; Available in PMC 2024 Jul 12. (PMC11241867; doi:10.15695/jstem/v7i2.10)
Supplement: Parisetal2024_AppendixD_Resources [file NIHMS2004214-supplement-Parisetal2024_AppendixD_Resources.pdf]

Inclusive Demographics in Biomedical Training – Paris, et al.
Appendix D. Resources

Resources and recommended readings for learning more about inclusive demographics.

PRIMARY TOOLS FOR OPERATIONALIZING LANGUAGE

REALD (Race, Ethnicity, Language, Disability)

This resource is validated and highly informative for how to phrase questions for various implementation settings (i.e., surveys, interviews, etc.). We highly recommend the implementation guide as a resource for thinking through demographics related to race, ethnicity, language, and disability, including how to report these demographic categories (McGee, 2020). A helpful flowchart for identifying approach can be found on page 58 of the Implementation Guide.

- Implementation Guide (McGee, 2020)

  McGee, M.G. (2020). Race, ethnicity, language and disability (REALD) implementation guide. Oregon Health Authority, Equity and Inclusion Division, Portland, Oregon. Retrieved April 11, 2023 from https://sharedsystems.dhsoha.state.or.us/DHSForms/Served/le7721a.pdf

- Quick guide to asking REALD questions and flowchart (Oregon Health Authority, 2020).

  Oregon Health Authority. (2020). Quick guide to asking REALD questions [Race, Ethnicity, Language and Disability (REALD)]. Equity and Inclusion Division, Portland, Oregon. Retrieved April 11, 2023 from https://www.oregon.gov/oha/EI/REALD%20Documents/REALD-Questions-Mini-Guide.pdf

SOGI (Sexual Orientation Gender Identity)

SOGI is an effort to increase and standardize sexual orientation and gender identity data collection across settings. The Oregon Health Authority (Oregon's statewide public health agency) has drafted recommendations with proposed questions to use when collecting SOGI data.

SOGI DRAFT Data Collection Recommendations (Oregon Health Authority, 2022)

Oregon Health Authority. (2022). OHA SOGI DRAFT Data Collection Recommendations. Retrieved April 11, 2023 [Updated November 2023] from https://www.oregon.gov/oha/EI/REALD%20Documents/DRAFT-SOGI-Recommendations.pdf

Website: https://www.oregon.gov/oha/EI/Pages/Demographics.aspx

START (STEM Assessment and Reporting Tracker)

This NIH-funded Science Education Partnership Award (SEPA) resource aims to help schools and STEM programs improve measurement of student development in STEM. START is curating approaches for inclusive evaluation of student populations to accelerate research on STEM inequities.

START webpage (Paris et al., 2021)

Paris, S.E., Lamkin, M., Alvord, T. W., and Marriott, L. K. (Eds.) (2021). STEM Assessment and Reporting Tracker. Oregon Health & Science University, Portland, Oregon, United States. Retrieved April 11, 2023 from https://www.startinstem.org

RECOMMENDED RESOURCES FOR LEVELING UP IN AN AREA

Framework and Guidance for Improving Equitable Practices in STEM

This comprehensive resource summarizes historical and contextual data, providing essential recommendations for improving training and diversity of our STEM workforce.

National Academies of Sciences, Engineering, and Medicine. 2023. Advancing Antiracism, Diversity, Equity, and Inclusion in STEMM Organizations: Beyond Broadening Participation. Washington, DC: The National Academies Press. Retrieved January 29, 2024 from https://doi.org/10.17226/26803

Iterative Demographics

Readers new to iterative approaches to demographics may find Krieger's (2012) framework helpful for understanding how populations are defined over time to promote health equity.

Krieger, N. (2012). Who and What Is a "Population"? Historical Debates, Current Controversies, and Implications for Understanding "Population Health" and Rectifying Health Inequities. The Milbank Quarterly, 90: 634-681. Retrieved January 29, 2024 from https://doi.org/10.1111/j.1468-0009.2012.00678.x

Race and Ethnicity

- The Government Alliance on Race & Equity (GARE) offers an informative website (2016) and communications guide (2018) for improving efforts. They offer racial equity tools and resources that can help programs advance opportunities for all.

  Government Alliance on Race & Equity (2016, October 18). GARE website. Retrieved April 14, 2023 from https://www.racialequityalliance.org/

  Government Alliance on Race & Equality (2018, May). GARE Communications Guide. Retrieved April 11, 2023 from https://www.racialequityalliance.org/wp-content/uploads/2018/05/1-052018-GARE-Comms-Guide-v1-1.pdf

- Consider approaches for how race and ethnicity data are reported (Schwabish & Feng, 2021)

  Schwabish J., Feng, A. (2021). Do no harm: Section 5 - The data behind the viz. Urban Institute. Retrieved January 29, 2024 from https://www.urban.org/sites/default/files/publication/104296/do-no-harm-guide.pdf

Disability

A NIH blog on "Enhancing Disability Inclusion" (Bernard, 2022) offers an inside look at enhancing disability inclusion, linking to the federal "Individuals with Disabilities Report" (2022).

Bernard, M. A. (2022). New recommendations for enhancing disability inclusion. National Institutes of Health [blog]. Retrieved April 14, 2023 from https://diversity.nih.gov/blog/2022-12-30-new-recommendations-enhancing-disability-inclusion

National Institutes of Health Advisory Committee to the Director (ACD) (2022). Working Group on Diversity (WGD) Subgroup on Individuals with Disabilities. Report. Retrieved April 14, 2023 from https://www.acd.od.nih.gov/documents/presentations/12092022_WGD_Disabilities_Subgroup_Report.pdf

Sex Minorities, Sexual Orientation Minorities, and Gender Minorities (SSGM)

We expect STEM programs to have the most questions about SSGM and how to inclusively incorporate measurement of SSGM populations while balancing student ages and program settings. The following resources are helpful for thinking about these areas and are great places to learn about how sex and gender terms have been used in research and where SSGM populations have been excluded or marginalized.

- The spectrum of sex and gender used in research (Morrison et al., 2021).

  Morrison, T., Dinno, A., Salmon, T. (2021). The Erasure of Intersex, Transgender, Nonbinary, and Agender Experiences Through Misuse of Sex and Gender in Health Research. American Journal of Epidemiology. Retrieved January 29, 2024 from https://doi.org/10.1093/aje/kwab221

- Ethical and safety concerns of minorities associated with SOGI data collection (Chase & Dinno, 2022)

  Chase, K. & Dinno, A. (2022). Oregon Health Authority Office for Equity and Inclusion Position Paper: Concerns about Safety of Minorities in the Collection of SOGI Data. OHSU-PSU

  School of Public Health Faculty Publications and Presentations. Retrieved April 14, 2023 from https://archives.pdx.edu/ds/psu/38511; [direct link: https://pdxscholar.library.pdx.edu/cgi/viewcontent.cgi?article=1509&context=sph_facpub]

- The current concept and issues on collecting transgender data with a great timeline of transgender terms and considerations(Kronk et al., 2022)

  Kronk, C. A., Everhart, A. R., Ashley, F., Thompson, H. M., Schall, T. E., Goetz, T. G., Hiatt, L., Derrick, Z., Queen, R., Guthman, E. M., Danforth, O. M., Lett, E., Potter, E., Sun, S. D., Marshall, Z., & Karnoski, R. (2022). Transgender data collection in the electronic health record: Current concepts and issues. Journal of the American Medical Informatics Association, 29(2), 271–284.

- Guidelines for supporting sex and gender equity (Heidari et al., 2016)

  Heidari, S., Babor, T. F., De Castro, P. et al. (2016). Sex and Gender Equity in Research: rationale for the SAGER guidelines and recommended use. Research Integrity Peer Review 1, 2. Retrieved January 29, 2024 from https://doi.org/10.1186/s41073-016-0007-6

COMMUNICATION TOOLS

If you are looking for a toolkit to discuss demographics or other issues with students, look to FrameWorks for approaches to various subjects, such as reframing adolescent development, community engagement, and mental health (FrameWorks Institute, n.d.).

FrameWorks Institute (n.d.). Tools and Resources. Retrieved April 11 from https://www.frameworksinstitute.org/tools-and-resources/

INTERACTIVE DATA TOOLS FOR EXPLORING DEMOGRAPHICS AND STEM EDUCATION

- The Condition of Education supports exploration of educational outcomes for students across training levels and demographic backgrounds (National Center for Education Statistics, n.d.). They offer yearly reports.

  National Center for Education Statistics (n.d.). Condition of Education: Annual reports on the condition of education. Retrieved January 29, 2024 from https://nces.ed.gov/programs/coe/ [static 2022 Report: https://nces.ed.gov/pubs2022/2022144.pdf]

  National Center for Education Statistics Annual Reports and Information Staff (n.d.). The Condition of Education [year] At a Glance [interactive data website]. Retrieved January 29, 2024 from https://nces.ed.gov/programs/coe/ataglance

- The National Center for Science and Engineering Statistics (NCSES) offers a data explorer, chart builder, and table builder that support education and employment of the STEM workforce, including how demographics intersect with STEM development.

  National Center for Science and Engineering Statistics (n.d.). Explore Data [interactive data website]. Retrieved January 29, 2024 from from https://ncses.nsf.gov/explore-data
